# Towards NetZero for hospital operating theatres

**DOI:** 10.1098/rsif.2025.0048

**Published:** 2025-09-10

**Authors:** Ian Eames, Anne Symons, Duncan Wilson, Yaman Rawas Kalaji, Lyndsay Muirhead, Jonathan Groome

**Affiliations:** ^1^ Department of Mechanical Engineering, University College London, London, UK; ^2^ Bartlett School of Sustainable Construction, University College London, London, UK; ^3^ Centre for Advanced Spatial Analysis, University College London, London, UK; ^4^ University College Hospital, London, UK; ^5^ The Royal London Hospital, London, UK; ^6^ Nuffield Health, Epsom, UK

**Keywords:** infection control, operating theatre, sustainability, NetZero

## Abstract

Hospital operating theatre suites are a particularly resource- and energy-intensive component of the health sector. Reducing their carbon footprint presents a significant challenge due to the necessity of maintaining patient safety. In this paper, we apply a multidisciplinary methodology to investigate and assess various strategies aimed at reducing the carbon footprint in hospital theatres. The strategies evaluated include (i) the duration of theatre ventilation operation, (ii) the efficiency of the ventilation strategy, and (iii) heat recovery systems and technologies. These approaches are assessed using a combination of theatre space monitoring (via building management systems), computational air-flow modelling and mathematical models. We develop a robust methodology that applies these modelling techniques to general hospital suites, enabling the estimation of reductions in CO_2_ equivalent.

## 1. Introduction

Callendar [1] was the first to postulate that the rise in atmospheric carbon dioxide (CO_2_) due to coal burning after the Industrial Revolution (from 292 ppm in 1898 to 310 ppm in 1936) could lead to a measurable increase in average temperatures in some regions of the world. Callendar developed a mechanistic model describing the integrated effects of incident sky radiation and radiated dark radiation (to use his phraseology), both of which depend strongly on latitude. Using this model, he linked an observed temperature increase of approximately 0.2°C at global weather stations to rising atmospheric CO_2_ levels and coal consumption. Callendar predicted a modest rise in global temperatures (0.39°C by 2000) based on emissions of 4.3 Gt CO_2_. However, he could not have foreseen the dramatic increase in hydrocarbon fuel consumption and anthropogenic carbon dioxide production, which reached 40.6 Gt CO_2_ in 2023, with atmospheric CO_2_ levels rising to 425 ppm in 2024. This escalation has contributed to an increase in average global temperatures by approximately 1.0°C above pre-industrial levels [2], a key driver of modern climate change. Other greenhouse gases, such as methane and nitrogen oxides (NOx), have significantly higher global warming potentials than carbon dioxide. These gases are primarily linked to the energy sector and agriculture.

The influence of an atmospheric temperature increase is felt through changes in weather patterns, especially in the Northern Hemisphere, due to the larger proportion of land mass in this space. Increases in atmospheric air temperature and extreme temperatures (both hot and cold) have led to more intense heat waves, increases in the size and frequency of fires and an increase in humidity, which lead to increased precipitation and flooding [3]. Climate change strongly influences public health through direct and indirect effects. Direct effects include heat-related morbidity and mortality and trauma from environmental hazards such as fire and extreme weather events. Indirect effects include the impact of loss events and food scarcity on the social determinants of health that widen pre-existing health inequalities.

Acknowledging the impracticality of a zero-emission strategy, a broader international NetZero challenge has been proposed. The goal is to achieve net-zero greenhouse gas emissions—typically measured in CO_2_ equivalent (CO_2_e)—by ensuring that emissions are offset by an equivalent amount of atmospheric carbon removal. This challenge is being addressed through coordinated efforts by governments, society and industry. In the UK, the government has enacted a legal mandate to achieve net-zero CO_2_ emissions by 2050, requiring a 100% reduction from 1990 levels [4]. Electricity and heat production account for the largest share of hydrocarbon consumption, followed by transportation. This challenge is being met through the growing activity of energy transition (with solar and wind farms forming the largest factor), increases in efficiency and technological advances. In the UK, global emissions have reduced from 590 to 430 Mt CO_2_e (per year) largely due to a greater fraction of renewables in electricity production and almost the elimination of coal (in 2022, less than 1% of electricity was generated by coal).

While the energy and transport sectors dominate carbon emissions, the healthcare sector also plays a crucial role in the NetZero challenge. Within each country, the health sector forms a significant and distinct part of the civil society and globally accounts for up to 5% of the world’s total carbon footprint [5]—more than aviation and shipping combined. The health sector typically employs 9–10% of the total workforce and, therefore, has a significant role to play in the NetZero journey; in the UK, the National Health Service (NHS) employs close to 1.5 million people and is responsible for 4% of UK emissions. A total of 62% of the NHS carbon footprint comes from the embedded emissions within our supply chain, with the remaining coming from energy, medical gases, travel, water and waste [6]. The NHS has set an ambitious target to be the world’s first NetZero health service with a target of 80% reduction of direct emissions by 2032 and NetZero for direct NHS emissions by 2040.

Hospitals primarily operate during daytime hours, with only critical areas—such as wards and emergency theatres—running continuously. Operating theatre suites, which consist of highly specialized rooms, are among the most resource-intensive spaces within a hospital on a per-square-metre basis. As a result, they contribute disproportionately to a hospital’s total CO_2_e emissions. In addition to the energy required to maintain cleanliness and thermal conditions in the theatre suites, surgical procedures themselves are resource-intensive, relying heavily on single-use instruments, drapes and gowns. Furthermore, anaesthetic procedures are surprisingly carbon-intensive due to the potent greenhouse gases present in volatile anaesthetic agents [7], highlighted the significant climate impact of these gases. Research by [5] identified anaesthesia as the largest contributor to the carbon footprint of operating theatres in Canada, the USA and the UK. Desflurane, a commonly used anaesthetic, has a global warming potential (GWP) 2540 times higher than CO_2_ (and 13 times higher than sevoflurane [8]). Phasing out desflurane—the largest contributor to operating theatre carbon emissions—would refocus attention on heating, ventilation and air conditioning (HVAC) systems, which account for 84% of the total footprint. Desflurane usage has dropped from 24% of total volatile anaesthetic use in 2018 to just 0.23% by 2024 [9]. Following this trend, NHS entities in England, Scotland and Wales, alongside the European Union, have moved to phase out regular use of desflurane [10]. Similarly, N_2_O, another potent greenhouse gas, is seeing reduced usage in anaesthetic practice. A landmark UK project on N_2_O management found that up to 99% of the N_2_O supplied to hospitals was lost before ever reaching patients [11]. As a result, the NHS is now recommending the closure of nitrous oxide manifold systems in favour of localized cylinder supply.

Rizan *et al.*’s [12] systematic review of the carbon footprint of surgical operations highlights that electricity is the largest source of greenhouse gas emissions, with a significant proportion attributed to HVAC. There are now numerous projects underway across health systems aiming to reduce emissions in theatre, focusing on anaesthetic gases, waste and the use of single-use items. Reducing energy use in theatres is essential for achieving large-scale decarbonization in healthcare. The use of Renewable Energy Guarantees of Origin (REGO) tariffs, on-site renewables and low-carbon power purchase agreements has yet to be widely adopted in healthcare. Additionally, it is important to focus on reducing energy demand sector-wide [13].

The challenge of achieving NetZero in theatre spaces, while maintaining patient safety and surgical efficacy, reflects the broader sustainability challenges facing the health sector. Addressing this issue requires an interdisciplinary approach, as it lies at the intersection of clinical practice, building design and thermal engineering. This paper aims to integrate expertise from architecture, engineering and clinical fields to develop a conceptual framework for reducing the carbon footprint of operating theatres, and to highlight exemplary cases where significant progress has been made.

The structure of this paper is as follows: first, the operating theatre suite, a collection of rooms designed to optimize air quality and people flow, is introduced. Section 2 reviews ventilation strategies and explains the fluid mechanical processes involved. While the validation of ventilation systems varies across countries, they generally fall into four main categories and the paper explores the equivalency between these methodologies. This comparison highlights the differences in ventilation efficacy and carbon burden. In §3, a methodology is developed for comparing the energy consumption of different systems. The design and operation of air-handling units are described, with particular focus on components related to thermal conditioning, which constitutes the largest portion of energy costs. Section 4 addresses improvements in the energy efficiency of theatre suites, breaking down the challenge into three key areas: suite design, ventilation systems and usage patterns. Finally, §5 illustrates the consequences of implementing these changes in terms of net costs and CO_2_ reductions, emphasizing their impact on the NetZero goal.

## 2. Operating theatre suite

### 2.1. Specialist areas

Theatre suites are specialist areas in healthcare facilities in which surgical procedures are performed and designed to be sterile and serviced with clean air that envelops the patient. The logistical challenge comes from the size of the surgical teams (which can vary from 5 to 15 staff for obstetrics) and the high thermal load caused by people, diagnostic equipment and thermal heaters. Ventilation systems in the UK are designed to deliver air at a temperature inthe range 18−23°C, with the relative humidity of less than 70%. Clinical staff have a local control of these temperature of these spaces, which are adjusted based on the patient age and the clinical procedure, with younger patients tending to need higher temperature [14] as they lose heat faster than adults.

**Figure 1.**
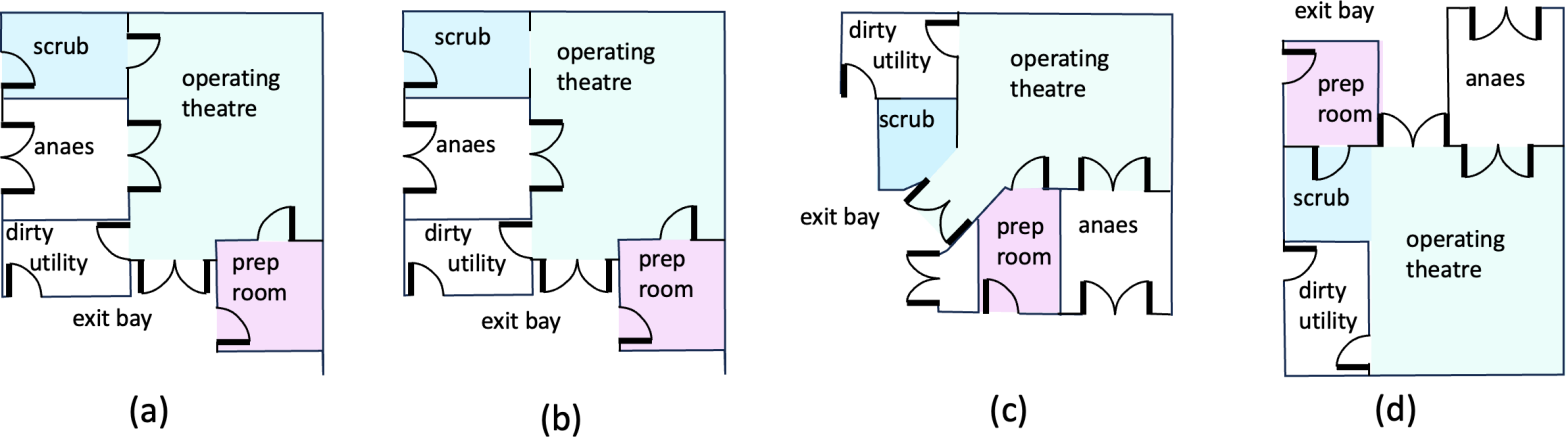
Different hospital suite configurations highlighted in Health Technical Memorandum (HTM) 03-01 and Health Building Note (HBN)-29. The typical configuration is (a) with the difference between (a) and (b) concerning the removal of the door between the scrub area and operating theatre. In (c), the scrub is an annex. In (d), staff pass through the preparation room to the operating theatre, through the scrub area.

**Figure 2.**
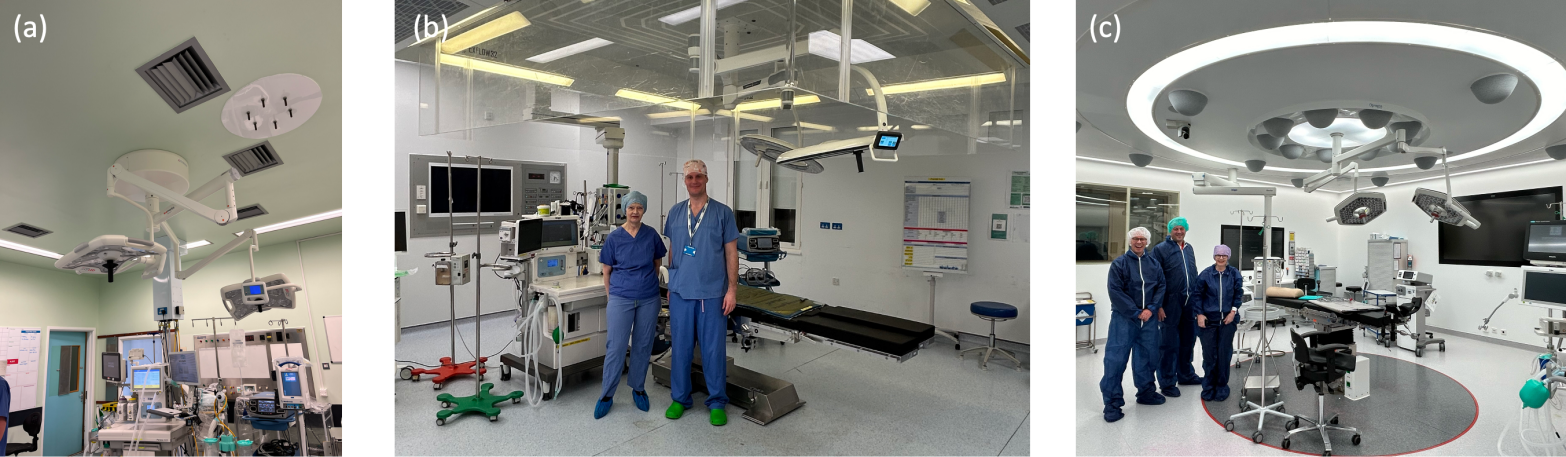
Photographs showing the different operating theatres: (a) turbulent flow ventilation (TFV) (or conventional ventilation), (b) laminar flow ventilation (LFV) (or ultra-clean canopy), and (c) thermally controlled ventilation (TCV). See table 1. In each case, the operating theatre is located in the middle of the room and the specialist ventilation highlighted. ((a) is taken by J. Groome, while (b,c) are taken by I. Eames.)

**Figure 3.**
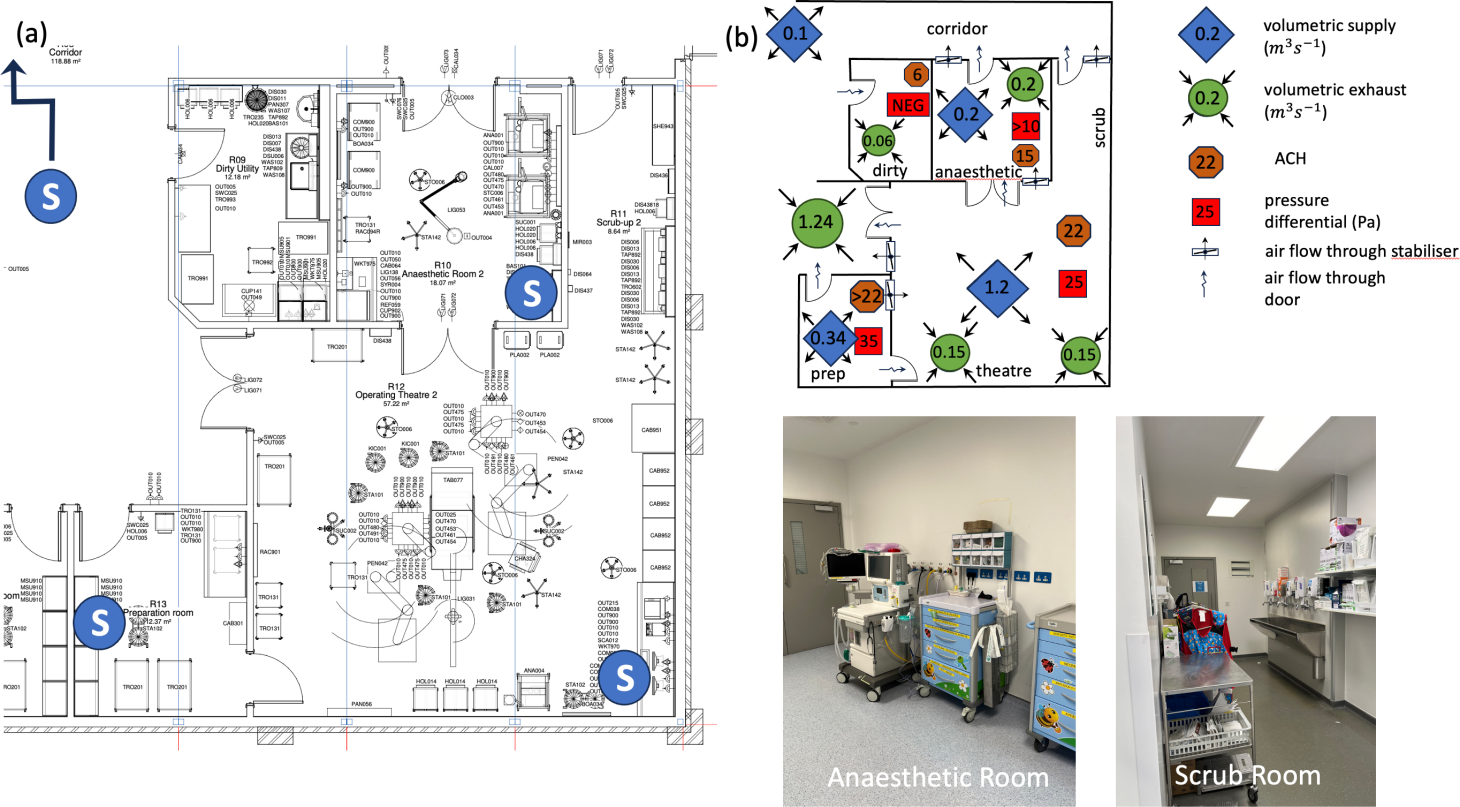
(a) Floor plan of a typical hospital operating theatre suite—Theatre 12 of Whipps Cross Hospital— incorporating parts of Theatre 11 on the left-hand side. The critical ventilation components are the volumetric supply and exhaust, with the design pressure differential (relative to the corridor) indicated. Pressure stabilizers are set between adjacent rooms to maintain the correct pressure differentials in the spaces. The location of the pressure sensors is denoted by S in (a). (b) Ventilation diagram for Theatre 12 suite is shown along with the volume flow rate, pressure differentials and air changes per hour (ACH). The volume flow rate for the operating theatre is $Q_{s}=1.2\;{\text{m}}^{3}\;{\text{s}}^{-1}$ because of the additional floor area for the scrub room. Photographs of typical anaesthetic and scrub rooms (taken at the Royal London), highlighting the duplicated equipment and the location of the sink.

Operating suites are a collection of rooms that comprise an operating room and a series of connected spaces to stage the level of cleanliness. There is wide variation of room configurations (see figure 1). For example, in the UK, the operating theatre suite typically consists of the following connected rooms/spaces: operating room, anaesthetic room, scrub room, preparation room, disposal/dirty utility and exit bay. figure 1a shows the plans for a typical theatre suite with the connected rooms and spaces labelled. The typical internal space within an operating theatre is shown in figures 2 and 3b. In Europe, the anaesthetic and scrub rooms may be absent, with the scrub area located in clean corridors to reduce the potential for aerosol transport from taps.

**Table 1.** List of terminology and alternative names for the three main ventilation systems, along with typical air changes per hour (ACH) and pressure differentials. Both laminar flow ventilation (LFV) and thermally controlled ventilation (TCV) can operate in an ultra-clean mode. The minimum volumetric flow rate of supply for an operating theatre ($Q_{s}$) are listed based on a theatre with floor area $A_{H}=55\;{\text{m}}^{2}$ and room height H=3 m . See also the typical values for ACH in table 1 from [20] and note the dependence on plenum size. A typical value for the total volumetric air supply and exhaust, to and from, a hospital suite for TFV and LFV is $Q_{Ts}=1.86\;{\text{m}}^{3}\;{\text{s}}^{-1}$ and $Q_{Te}=1.73\;{\text{m}}^{3}\;{\text{s}}^{-1}$, respectively. For TCV, typical values are $Q_{Ts}=2.83\;{\text{m}}^{3}\;{\text{s}}^{-1}$ and $Q_{Te}=2.36\;{\text{m}}^{3}\;{\text{s}}^{-1}$ .

description (alternative names)	acronym	ACH	$Q_{s}$ (${\text{m}}^{3}\;{\text{s}}^{-1}$)	ΔP (Pa)	mode of operation
turbulent flow ventilation	TFV	22	1.0	25	full (100%)
conventional ventilation					setback (∼40%)
turbulent mixed ventilation (TMV)					
laminar flow ventilation	LFV	22−65	2.98 (for 2.8 × 2.8 m^2^)	25	full (100%)
ultra-clean ventilation (UCV)		22−85	3.89 (for 3.2 × 3.2 m^2^)		conventional (no fans)
laminar air flow ventilation (LAF)					setback ( ∼40% )
temperature-controlled ventilation (T ${,}_{e}$ AF)	TCV	42	1.9	25	variable (5% to 100%)

Operating theatre suites were originally designed on the principles of ‘clean and dirty’ corridors, which was the preferred concept prior to 1968 when it was considered acceptable to ‘bag’ dirty goods and waste and transport them through the clean corridor. This has led to the two-corridor system, still in operation today—an outer corridor where staff and patients enter the department before staff change into ‘scrubs’ and enter into the ‘clean’ zone. In [15], a door or hatch led from the theatre into a ‘dirty zone’ rather than an actual room. The removal of the dirty corridor introduced the ‘Dirty Utility’ as an additional room and part of the suite where waste could be stored during operating sessions and taken out through the clean corridor once the operating sessions had finished. The specific role of each space is briefly reviewed.

In the UK, the The Health Technical Memorandum for specialist ventilation in healthcare premises [16] (commonly referred to as HTM-03) outlines ventilation requirements and room configurations, with particular emphasis on operating theatres. Complementing this, the Health Building Notes (HBNs) provide best-practice guidance for designing and planning modern healthcare facilities. Originally introduced in 1967, HBN-26 has undergone periodic revisions to incorporate advancements in medical technology, evolving models of care and the increasing imperative to reduce the carbon footprint of healthcare environments. Considering these factors, this study aims to reimagine the purpose and functionality of various rooms within operating suites. Below, we outline the functions and roles of these spaces, laying the foundation for a more efficient and sustainable approach to healthcare design.

— *Operating room/theatre*. This is the primary space where surgical procedures are performed and is the cleanest area in a hospital. Since 1962, when the first ‘Ultra Clean Ventilation’ system was installed at Wrightington Hospital, the term 'ultra-clean’ has been used to designate the cleanest zone within the operating room. Three teams operate within the theatre, each occupying distinct zones: (i) the surgical team, including surgeons and scrub nurses; (ii) the anaesthetic team, comprising anaesthetists and operating department practitioners or anaesthetic nurses; and (iii) the support team, consisting of circulating nurses and healthcare assistants. While all staff wear scrubs, only the surgical team wears gowns over their scrubs and ‘scrubs in’ at the scrub-up area before entering the operating room. Scrubs are the standard attire for personnel working in the ‘clean’ zone of the operating department.
— *Anaesthetic room*. In the UK, most patients are anaesthetized in this room before being transferred to the operating theatre. Patients enter from the clean corridor and are prepared for surgery in this space. Typically covering approximately 20 ${\text{m}}^{2}$, the anaesthetic room represents a significant footprint, often resulting in the duplication of expensive equipment and infrastructure already present in the operating room. While this practice is standard in the UK, during high-risk operations and the COVID-19 pandemic, patients were frequently anaesthetized directly in the operating room. The necessity of separate anaesthetic rooms remains a topic of academic discussion, weighing their benefits against efficiency concerns. Notably, this practice is uncommon in much of Europe and the United States.
— *Scrub room/area*. This is where the surgical team gowns after scrubbing their hands and lower arms. There are four common configurations for scrub rooms, as illustrated in figure 1. In examples figure 1a,b, the scrub area is enclosed, with the latter featuring a connecting door to the theatre. In figure 1c, the scrub area is integrated into the operating room, requiring staff to enter through the ‘patient exit’ to access the scrub sink. In figure 1d, the scrub area is also within the operating room, but in this layout, the preparation room is accessed through the scrub area. These configurations contrast with European and US theatre suites, where the scrub area is typically located in the clean corridor outside the operating theatre suite. Designs incorporating a door between the scrub area and theatre reduce the risk of waterborne infections transmitted via aerosol sprays, while placing the scrub in a corridor can lower the overall volumetric air flow through the theatre suite.
— *Preparation room*. This is where sterile surgical packs are delivered and prepared before surgery. Supplies enter from the clean corridor, are unpacked and are made ready before being transferred into the operating room. This enclosed room maintains the same air change rate as the operating theatre and appears to be the only consistently present space within the operating theatre suite.
— *Dirty utility and disposal facilities*. The requirement for a dedicated dirty utility room in each theatre suite is not mandatory, and its necessity remains somewhat ambiguous. Early guidance from 1967 outlined a three-corridor system: (i) an outer corridor for staff and patient entry, (ii) a clean corridor providing access to the operating theatre suites, and (iii) a disposal corridor at the rear of the theatre suites. Operating rooms featured either a hatch into this corridor or a door into a holding bay. By 1968, theatre disposal items were placed in bags and removed directly from the operating room, eliminating the need for a dirty corridor. Subsequent theatre suite designs included a dirty utility room accessible from both the operating room and the clean corridor, allowing dirty items to be stored until the end of a theatre list and providing facilities for between-case cleaning. Some configurations allow for shared dirty utility rooms between multiple theatre suites, optimizing space and resource usage.

Table 2 shows a comparison across different countries of the ventilation requirements of hospital theatres. In European and US theatres, the scrub area is situated outside the operating room and reduces the potential for waterborne infections to contaminate either the operating or preparation areas. This would then focus the theatre suite design to be a combination of an operating room and a preparation room, which with hermetically sealed interlocking doors would not only provide cleaner air quality but would reduce energy costs.

**Table 2.** Standards and requirements for theatre ventilation across countries.

country	standard	year	sound	ACH	ΔP	LFV	filtration	**RH (%)**	tempT	CFUs
			intensity (db)		(pa)	velocity			(°C)	( ${\text{m}}^{-3}$ )
						( $\text{m}\;{\text{s}}^{-1}$ )				
UK	HBN 26/ADB	2013		25	+25		F7	35−60	18−25	
	HT03-01: TF	2023	48	>22			EU10	35−70	18−25	
	HT03-01: LAF	2023	53	>22		0.2−0.38	EU11	35−70	18−25	<10
Australia	AS 1668:2	2024		>20				35−70	18−27	<29
Austria	ONORM H 6020	2007	45		+	0.22−0.45	F7+H13	35−45	20−24	
Chile	ASHRAE 170	2017	40−50	>20			MERV7+MERV14	45−60	18−24	
Finland	ISO 14644-1	2015	45−55		+15				21−23	
France	NF S90-351	2013	28		+15	0.25−0.35	F6+F7+F13	45−55	19−26	<20
Germany	DIN 1946-4:2018	2018	45	>23		>0.33 m³ s^−1^	M5+F7+F9+H13	30−50	19−26	4 to 7
	standard VDI 2167		48			0.24−0.30	M5+F7+F9+H13	30−50	20−25	
The Netherlands	FMS	2022		>20	≥5		ePM1 90 %/H13	<65%	18−23	10 avg
	NOV	2022								(30 max)
	VCCN Guideline 7	2017								
Sweden	SIS-TS 39:2015	2015	<45	N/A	≥5	N/A	F7/F9/ ≥ H14	<70%	22 ± 4	<5 avg
			(not in TS39)							(15 max)
Switzerland	SWKI 99-3	2003	48			0.23−0.25	F7+F9+H13	30	19−26	<10/50/200
US	ASHRAE 170	2017	40−50	>20		0.23−0.25	MERV7+MERV14	20−60	20−24	<10/50/200

### 2.2. Ventilation strategies

The potential for nosocomial infection is raised by a surgical procedure, particularly those that expose deep tissues for long periods of time [17]. Even in a sterile environment, the potential for infection is greatly raised in circumstances when biological matter and fluids become aerosolized during cutting, cleaning, drilling (see [18]), or skin shedding from clinical staff. The most significant challenge occurs in orthopedics or neurosurgery due to the mass of potential aerosolized material that may enter the air and the complexity of the surgery (meaning large surgical teams), which explains why this discipline was the first to propose the introduction of ultra-clean ventilation [19].

The ventilation strategy depends on the type of operation performed and the risk and consequence of a surgical site infection. Figure 1b shows a ventilation plan for a hospital theatre suite. The air supplied to, and exhausting from, a theatre suite is set to maintain clean air within each room, an air flow path (from the most sterile to the least sterile spaces) and the pressure differential between adjacent rooms. The supply and extract air flows rates (in ${\text{m}}^{3}\;{\text{s}}^{-1}$) are $Q_{s}$ and $Q_{e}$, respectively. The typical volume flow rates through a single hospital suite are listed in table 1.

**Figure 4.**
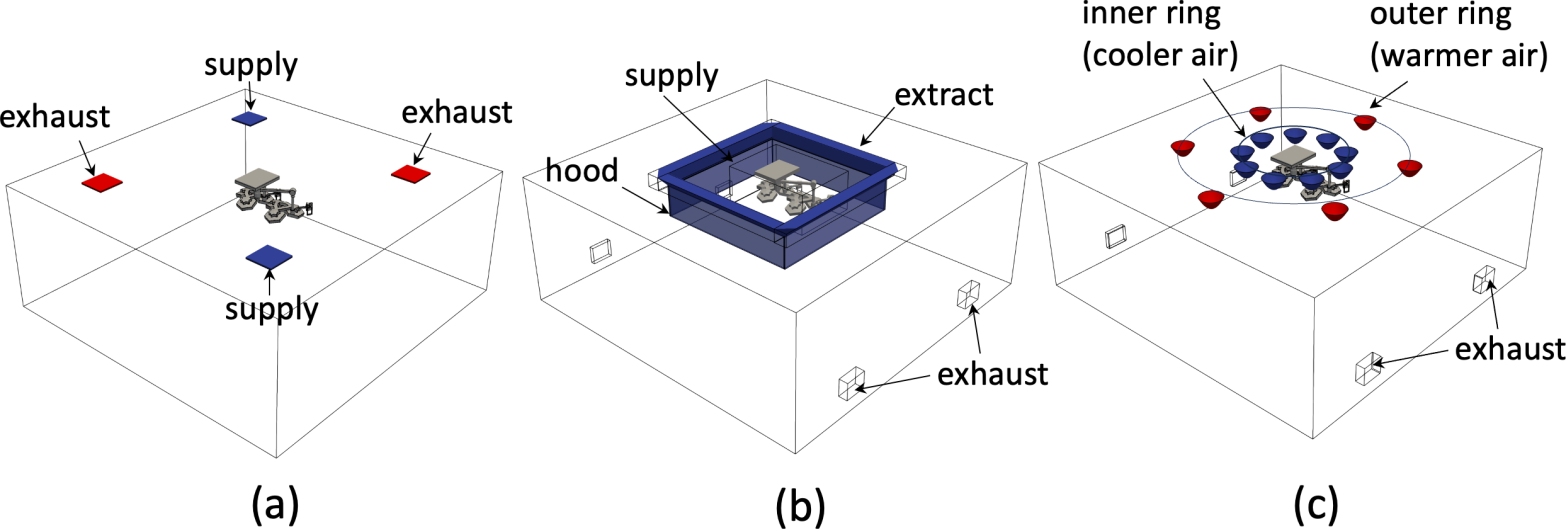
Schematic of the computational domains used to explore the critical flow physics with (a), (b) and (c) corresponding to TFV, LFV and TCV. The room height H=3 m, while the floor area is $A_{H}=55\;{\text{m}}^{2}$. The outward normal from the supply and extract surfaces is $u_{n}$. In (a), $u_{n}=1.4\;\text{m}\;{\text{s}}^{-1}$ on the supply vents, (b) $u_{n}=0.38\;\text{m}\;{\text{s}}^{-1}$ on the supply hood and $u_{n}=-0.6\;\text{m}\;{\text{s}}^{-1}$ on the extract hood and (c) $u_{n}=0.5\;\text{m}\;{\text{s}}^{-1}$ over each diffuser. A uniform pressure constraint is applied on the exhaust surfaces.

The preparation room is designated as the cleanest space within the suite, with a flow cascade driven from the theatre to the corridor and then onto the dirty utility through a pressure cascade. The cascade flow occurs because $Q_{s}>Q_{e}$ in all rooms (except the dirty utility) where air is extracted. The direction of the excess volume flux, as well as the proportion that passes through door gaps and pressure stabilizers, is specified in the ventilation plan (figure 3a); the pressure stabilizers open at a set pressure differential and maintain a fixed pressure difference. The ventilation strategies are classified in terms of the levels of turbulence and buoyancy effects and fall into three types listed in table 1 and shown in figure 2. To illustrate the flow physics, a series of numerical simulations were performed on a single square room with floor area $A_{F}=55\;{\text{m}}^{2}$, room height H=3 m. The room geometry, supply, extract and exhaust vents used in the computational simulations are shown in figure 4. The simulations were based on standard finite-volume model of a turbulent flow, described in the appendix, with additional models for the thermally controlled ventilation (TCV) to account for thermal physics. The simulations were run for 120 s, so the flow became established and the flushing and statistics were analysed in the ensuing period.

**Figure 5.**
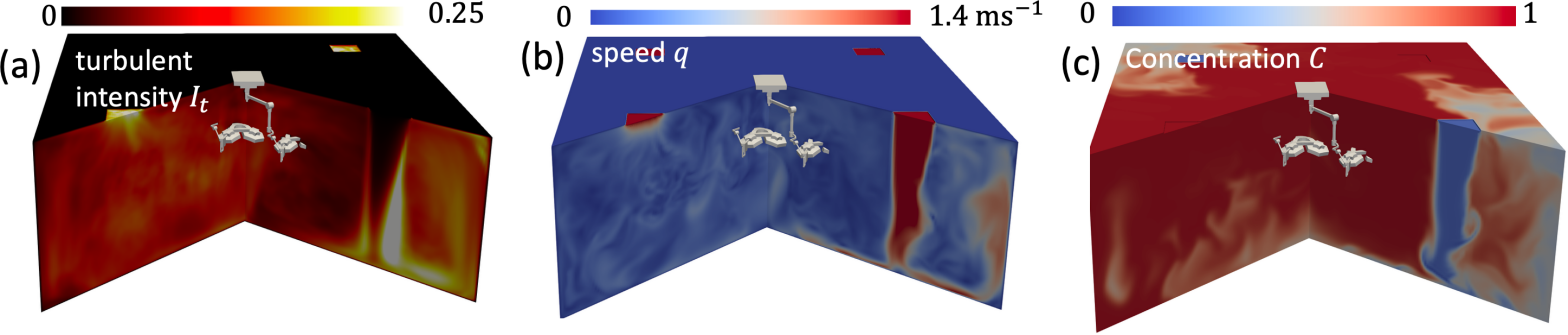
Numerical results showing the flow characteristics of TFV. The turbulent intensity ($I_{t}=u_{\text{rms}}/1.4\;\text{m}\;{\text{s}}^{-1}$) and mean flow (q) are shown in (a,b) respectively. The flushing of a passive contaminant, introduced after flow is established, is shown in (c).

Figure 2a shows a typical turbulent flow ventilation (TFV) system (Whipps Cross Hospital) where the supply air creates an unsteady flow, generating mixing within a closed space, leading to rapid dilution of an airborne contaminant. Approximately $0.3\;{\text{m}}^{3}\;{\text{s}}^{-1}$ of the air is removed from an exhaust within the space, and the remainder discharges through pressure stabilizers and undercut doors (figure 3b). The grille on the supply side is used to promote small-scale turbulence with a high turbulent intensity, which rapidly decreases in intensity and creates a room-scale flow with a lower turbulent intensity. The narrowness of the supply grille compared with the room height means that the turbulent jet created quickly disperses. Dilution and mixing tend to be associated with the straining regions between the vortical structures [21]. The vortices that occupy the central region of the room tend to be larger than the size of the inlet and their movement leads to ballistic dispersion. A lateral sweeping air flow (from the unsteady flow) serves to remove air from the vicinity of the patient. The type of exhaust grille, and indeed their size, has a weak effect on mixing except for influencing the characteristics of the mean flow in the region. This example of strong turbulence within low-aspect-ratio spaces is similar to the channel flow turbulence, where the kinematic effect of the floor and ceiling generates an integral scale that increases approximately linearly from the bounding walls. Figure 5 shows a numerical simulation with two supply and exhaust regions ($Q_{e}=Q_{s}=1.0\;{\text{m}}^{3}\;{\text{s}}^{-1}$). The turbulent intensity is largest near the shear layer created by the air supply and wall, and tends to be uniform within the central space with wakes around the lights. The flow near the surgical lamps is unsteady so that a stable wake is not created (figure 5b). After the flow is established, the room is then initialized with a passive contaminant (figure 5c), which is mixed by the turbulence and swept out of the space.

**Figure 6.**
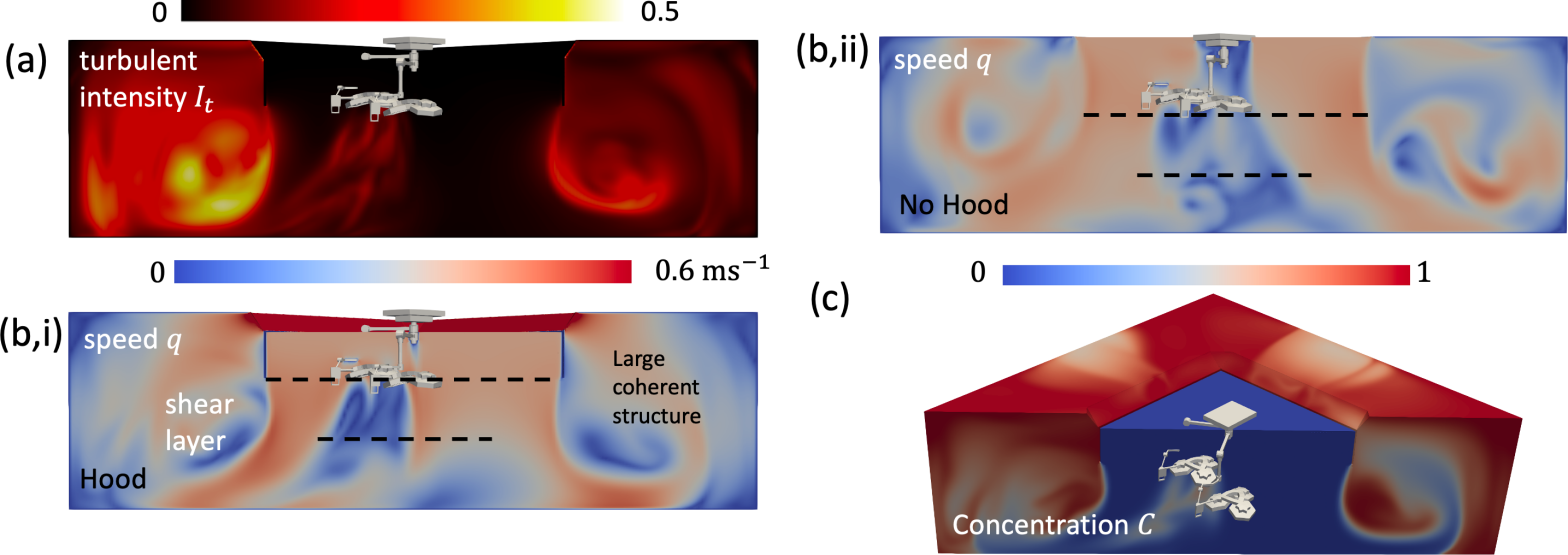
Flow characteristics of a laminar flow ventilation (LFV) system, operating in an ultra-clean mode, are shown, highlighting the critical features of the laminar flow, irrotational straining region and interfacial shear layer with the recirculating regions at the side. In (a), the turbulent intensity is shown ($I_{t}=u_{\text{rms}}/0.38\;\text{m}\;{\text{s}}^{-1}$) in a plane that runs diagonal across the domain. The instantaneous flow speed (q) is shown in (b,i) for the case of a canopy hood present, while in (b,ii), the extract and hood are absent. The concentration field is shown in (c) t=20 s after the flow is established.


Figure 2b shows a typical laminar flow ventilation (LFV) system (The Royal London Hospital) where a uniform vertical flow is introduced over a large square area ($3.2\times 3.2\;{\text{m}}^{2}$) above the patient and the supply is surrounded by a curtain extending 2 m above the ground. The air flow rate from the air handling unit (AHU) into the theatre is enhanced by four fans that supply air (which creates an additional 63 air changes per hour (ACH)—see table 1) from a peripheral extract to increase the canopy flow (see figure 6b,i), recycling 74% of the air in the space. The inner region beneath the canopy, which forms about 36% of the total planform area, is the ultra-clean space. The descending air, which passes through a high efficiency particulate air (HEPA) filter and a fine porous sheet, is characterized by a low level of turbulence. The ventilation is effective because the path from the air supply to the patient is direct—a form of displacement ventilation beneath the hood—rather than working on the principle of dilution mediated through turbulence. The descending flow is characterized by an intense shear layer generated by the sharp edge of the canopy hood. The kinematic constraint imposed by the ground leads to a widening of the inertially dominated strained flow. The centreline vertical velocity decreases from the value at the bottom of the curtain (height h=2 m above the ground) to zero on the wall, with the decrease approximately

(2.1) $$\begin{array}{lcr} & u_{z}∼-U_{I}\frac{z}{h}, & \end{array}$$

where z is a distance from the ground (see [[22], equation 2.7.11], for two-dimensional and axisymmetric flows) and $U_{I}$ is the vertical downwards velocity at z=h. The edge of the shear layer has a width $y=w/2(h/z{)}^{n}$ where w is the canopy width/radius and n typically varying between 1 and 2 (the limits for a slender rectangular and circular inlet, respectively). Figure 6a shows the numerical results of flow beneath a square canopy of width 3.2 m, with the flow exhausting through four low-level extracts. The flow within the canopy is characterized by low levels of turbulence (figure 6a), with the outer region characterized by flapping shear layers. The canopy width is comparable to both the room width and height, with confinement effects leading to a slower reduction of $u_{z}$ with z. The ceiling level extract flow, located at the perimeter of the canopy, serves to widen the shear layer, while room confinement tends to confine the shear layer flow (figure 6b,i) and leads to the creation of a recirculating region between the canopy and walls. Several recent studies, notably Duque-Daza *et al.* [23], have used computations and measurements to confirm the presence of the recirculating region and potential disruption caused by the presence of staff and equipment. The absence of a confining hood and flow extract leads to a much more disrupted and unsteady shear layer (see figure 6b,ii) and a more extensive wake around the surgical lamps. Figure 6c shows the flushing of an airborne contaminant by the air supply, confirming the influence of lights in increasing the local residence time and also the disruption of the recirculating region in outer space.

**Figure 7.**
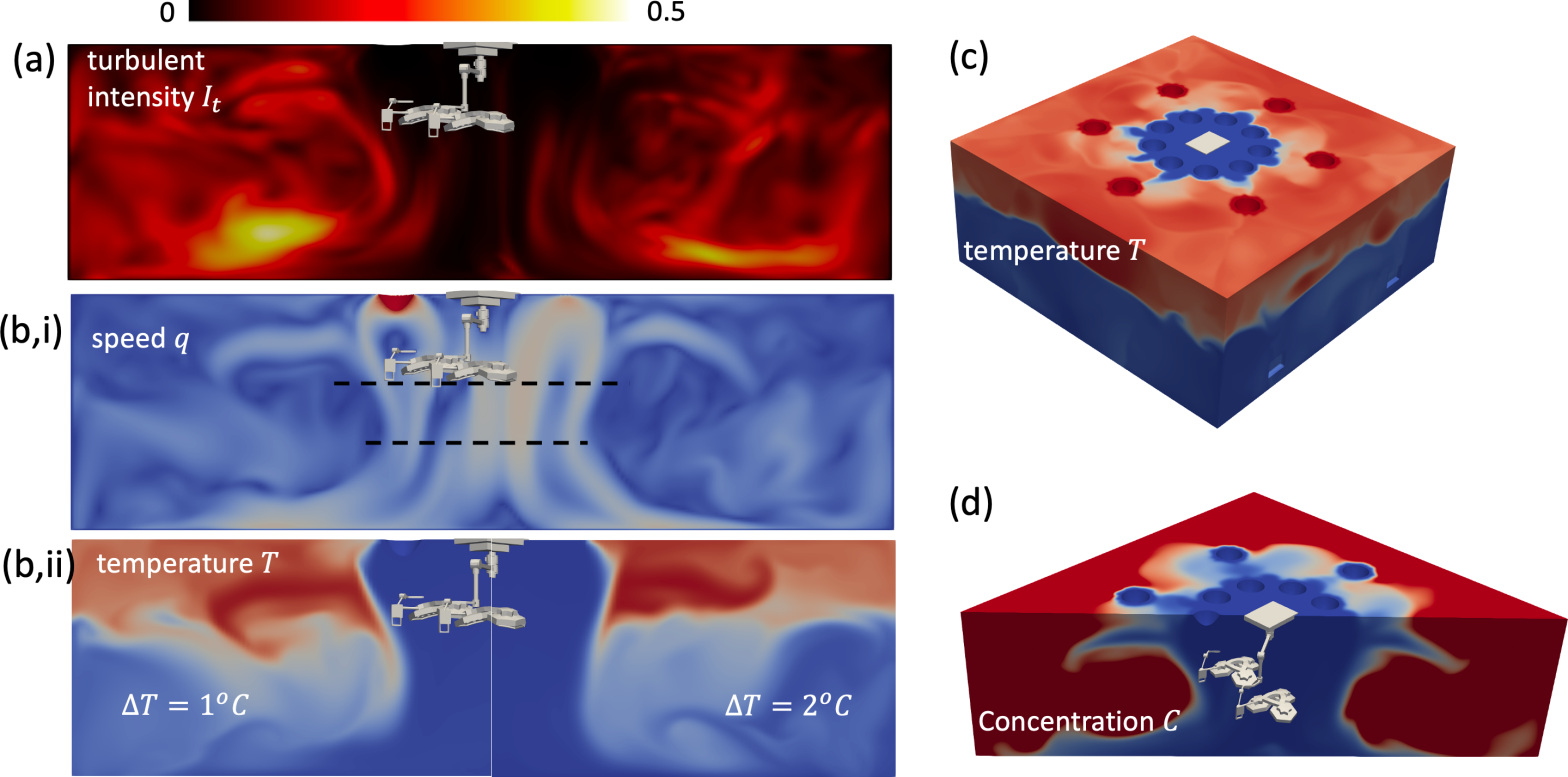
Flow characteristics of a temperature-controlled ventilation system (TCV) for a temperature differential of $\Delta T=2^{\circ }\text{C}$. In (a) the turbulent intensity ($I_{t}=u_{\text{rms}}/0.5\;\text{m}\;{\text{s}}^{-1}$) and (b,i) mean speed (q) is shown in a plane that runs diagonal across the domain. In (b,ii), the influence of reducing the temperature differential (comparing $\Delta T=1^{\circ }\text{C}$ with $2^{\circ }\text{C}$) on the thermal stratification is shown; the temperature field on the top and sides of the domain are shown in (c) for $\Delta T=2^{\circ }\text{C}$. The concentration field C is shown at a time t=20 s after the flow has been established and the supply air is introducing air with C=0.

Figure 2c shows a typical TCV system (Sint Maartenskliniek Hospital). Clean air is introduced via ceiling-mounted inverted domed diffusers, arranged in concentric circular configurations, with low-level extraction. The room is separated into a circular inner ultra-clean region and an outer region. Figure 7 shows the typical air flow and thermal field associated with a TCV. Figure 7a shows that, similar to LFV, the inner region is characterized by low levels of turbulent intensity, but differs in the outer region, where the turbulent intensity is more uniform for TCV. The temperature differential between the inner and outer ring of diffusers is essential for maintaining the ultra-clean inner region, with the inner vertical cold shower partially driven by negative buoyancy and the air accelerating as it descends (see figure 7b,i). Figure 7b,ii shows the vertical stratification of temperature within the space and its importance for confining the flow from the inner region, and that this feature is still persistent when the temperature differential is reduced from $\Delta T=2^{\circ }\text{C}$ to $\Delta T=1^{\circ }\text{C}$. The elevated view of the thermal field (figure 7c) shows that the stratification is persistent across the room. The temperature differential between the inner and outer diffuser rings means that they play slightly different roles in cleaning the air. As evident in figure 7d, cool clean air from the inner rings descends and displaces room air from around the patient, while the warmer clean air, from the outer rings, tends to mix with the air in the upper stratified layer.

**Figure 8.**
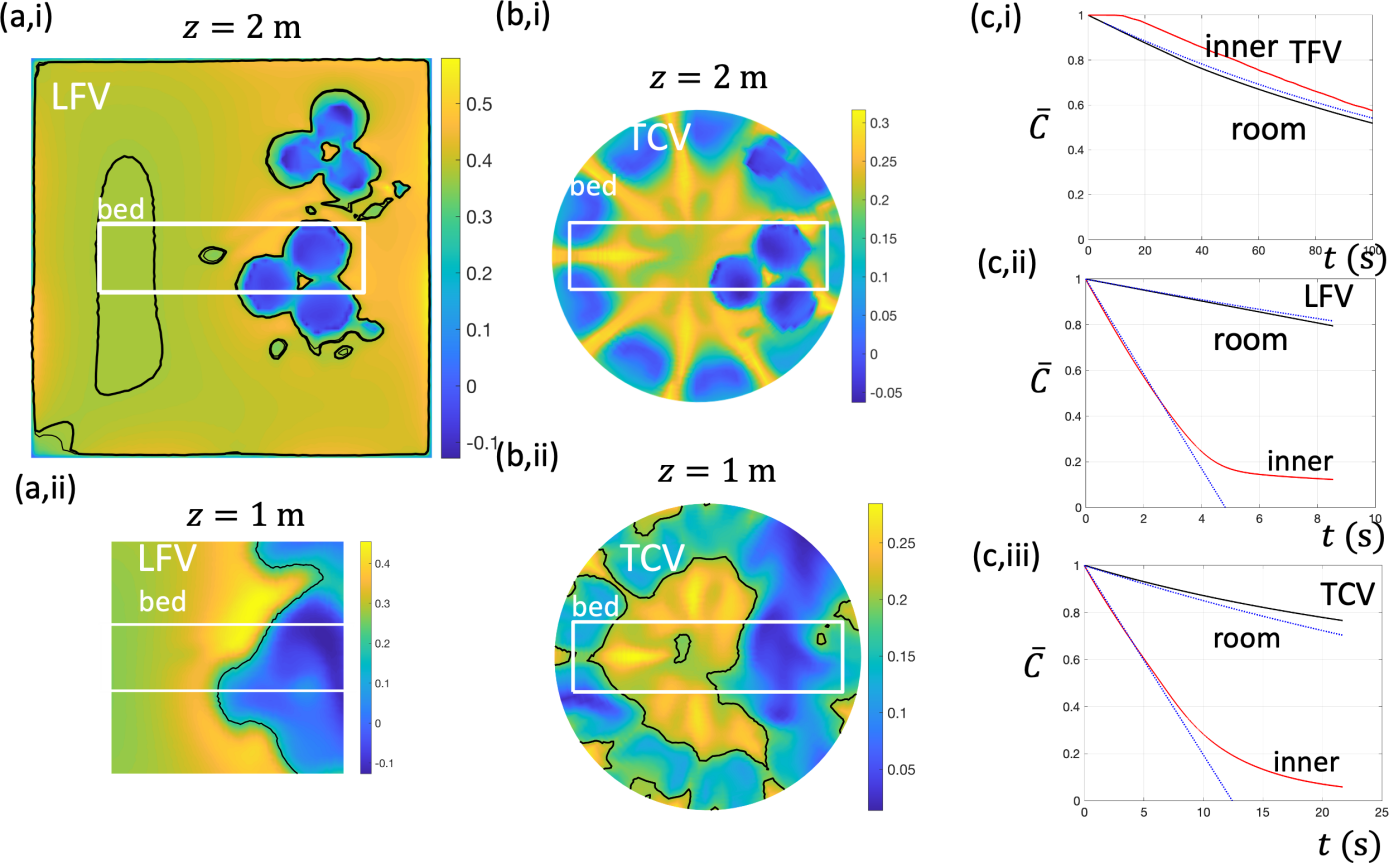
Time-averaged vertical (downwards) air velocity, −uz, at z=2 m and z=1 m for (a) LFV and (b) TCV. In (a) the span is the canopy width and inner region ( approx. 60%. of the canopy width) and (b) the inner region of radius 1.1 m. The outline of a theatre bed (length 2.1 m, width 0.56 m) gives a representative lengthscale. In (c), the decrease in the concentration of a passive contaminant is shown as a function of time for the whole room (black curve) and inner volume (red curve) for (i) TFV, (ii) LFV and (iii) TCV. The inner volume extends from 1 m to ceiling height in a square of circular region (width 1.92 m or radius 1.1 m, respectively). The blue curves are theoretical predictions based on perfect mixing (2.3) or flow displacement (2.4) and applied to the whole room (*V_r_*) or inner region (*V_r_*).

Figure 8a,b show the time-averaged vertical downwards air velocity (−uz) at z=1 m and z=2 m for LFV and TCV, respectively. The location and dimensions of a typical theatre bed are highlighted. The isocontour for ${\bar{u}}_{z}=-0.2\;\text{m}\;{\text{s}}^{-1}$ is highlighted in figure 8a,b,ii. The surgical lamps create a wake behind them, which is evident for z=1 m, which lengthens the residence time of material within these spaces. The results indicate that buoyancy effects in TCV tend to suppress the recirculating region behind the lamps, which is evident in LFV.

The potential risk associated with an airborne hazard is related to its concentration within a specific space. This can be assessed by analysing the displacement from, and dilution within, a prescribed volume of passive material, initially uniformly spread through the theatre (with concentration $C_{0}=1$), just as Wells [24, p. 345] examined in assessing the risk posed by airborne infection. Defining C(x,t) as the scalar concentration at a point, the evolution of the average concentration C within a specific volume $V_{s}$ changes according to


(2.2) $$\frac{\text{d}(V_{s}\bar{C})}{\text{d}t}=S_{c}-\int_{\text{inlet + outlet}}Cu\cdot \hat{n}\text{d}S,$$

due to the flux through the boundaries of the volume and $S_{c}$ is the total volumetric source. When the contaminant is well-mixed; in a whole room (volume *V*_r_) the average concentration on the exit surface $C_{i}$ is approximately the average room concentration, the average room concentration decreases as


(2.3) $$\bar{C}(t)=C_{0}\exp\left(-\frac{Q_{r}t}{V_{s}}\right),$$

where the rate of removal of air from the room is $Q_{r}$. The difference between the rate of air removal from a room and the volumetric air supply, $Q_{s}$, tends to be small. Equation (2.3) This is the standard Riley model for the decrease in contamination concentration [25] and a reasonable approximation providing stratification is not important. Ultra-clean ventilation (LFV and TCV) is characterized by a displacement flow in the vicinity of the patient so that the mean concentration within this space decreases (from $C_{0}$) due to a displacement effect ($C=C_{0}$ on the exit surfaces), i.e.


(2.4) C(t)=C0(1−QistVs),

where the rate at which air is added to the inner volume $V_{s}$ space is $Q_{is}$. The flushing of material from the entire room and inner region was assessed for the three ventilation strategies. The inner regions were defined as extending from 1 m above the ground to the ceiling and formed a block of width 2.2 m for LFV and TFV and a cylinder of radius 1.1 m for TCV. Figure 8c shows the decrease of the average concentration with the room and inner space, evaluated numerically. The simulations confirm the decrease for the room is reasonably predicted by the exponential decrease (2.3). The displacement ventilation is evident in figure 8c,ii, c,iii as a consequence of the inflow of clean air. For LCV, the vertical air flow is approximately vertical and uniform over the entire volume with the deviation from the linear decrease (2.4) due to the kinematic effect of the ground causing the flow to slow down (e.g. (2.1)). For TCV, the vertical flux of air through the inner region is approximately the fraction of air discharged into $V_{s}$ or $Q_{is}=\lambda_{h}N_{h}Q_{h}$, where $N_{f}=9$ is the number of inner hoods and $Q_{h}$ the volume flux through one hood. Figure 8c,iii shows a comparison between the computations and (2.4) for $\lambda_{h}=0.4$. The reduction in C can be largely understood from an assessment of the local flow fields explained by the total volume flow of clean air entering from the hoods into the inner region (giving λ_*h*_ ≈ 0.5).

### 2.3. Ventilation validation

The ventilation systems and air handling units that supply the operating theatre suites of the hospital must be validated annually. The validation techniques can be placed into four main groups, and each country will place a greater emphasis on one technique. Four techniques are:

(a) *Integral constraint.* This criterion is based on defining a total volumetric supply into a space through ACH,
(2.5) $$\text{ACH}=\frac{3600Q_{s}}{V_{r}},$$
  where $V_{r}$ is the volume of the empty room. Table 2 describes the different standards between different countries. In the UK, HTM 03-01 specifies that ACH≥22 (HTM 03-01 appendix 7); in the US [26], the ACH≥20, with a minimum of 20% fresh air.
(b) *Kinematic constraint.* This criterion is typically applied to LFV. The requirement is based on distinguishing between the space beneath the whole canopy ($S_{C}$) and inner clean zone ($S_{I}$). The kinematic constraints are based on a time average of the vertical velocity field, $u_{z}$, where
(2.6a,b) $${\bar{u}}_{z}(x,y\in S_{O},z=2\;\text{m})<-0.38\;\text{m}\;{\text{s}}^{-1},\;\min({\bar{u}}_{z})(x,y\in S_{I},z=1\;\text{m})<-0.2\;\text{m}\;{\text{s}}^{-1},$$
  (HTM 03-01, §8.97). Table 2 contrasts the different standards between countries. In addition, since the canopy is usually fed by multiple fans, the average over each unit should not exceed 6% of the average over the whole canopy. The size of the clean zone depends on the type of surgeries performed with circular region of 1.4 m for local surgeries and 2.8 $\times \;2.8\;{\text{m}}^{2}$ or 3.2 $\times \;3.2\;{\text{m}}^{2}$ for major orthopedic procedures. Taking the integral over the air flow at a height of 2 m gives a minimum volume flux of 2.98 ${\text{m}}^{3}\;{\text{s}}^{-1}$ (ACH=65) or 3.89 ${\text{m}}^{3}\;{\text{s}}^{-1}$ (ACH=85) for a typical operating theatre.
(c) *Particle loading*. This criterion is sometimes called the entrainment test and involves introducing challenge air (air with particles) at a height 2 m on the outside edge of the canopy (or on the shear layer if the canopy is not present). The challenge air flow is set to be the same as the average vertical velocity, with the sampler located at a height of 1 m. The test is that no measurements are less than 10% of the challenge at each test position in the outer zone and less than 1% of the challenge, within the inner zone.
(d) *Colony forming unit load*. This is a microbiological sampling approach based on air sampling in specific areas of the space. Ultra-clean air is defined as having less 10 CFU ${\text{m}}^{-3}$ (colony forming units) present at the wound site during a surgical procedure. In practice, levels of only 1 CFU ${\text{m}}^{-3}$ are often attained (see table 2 for a comparison between different countries).

**Figure 9.**
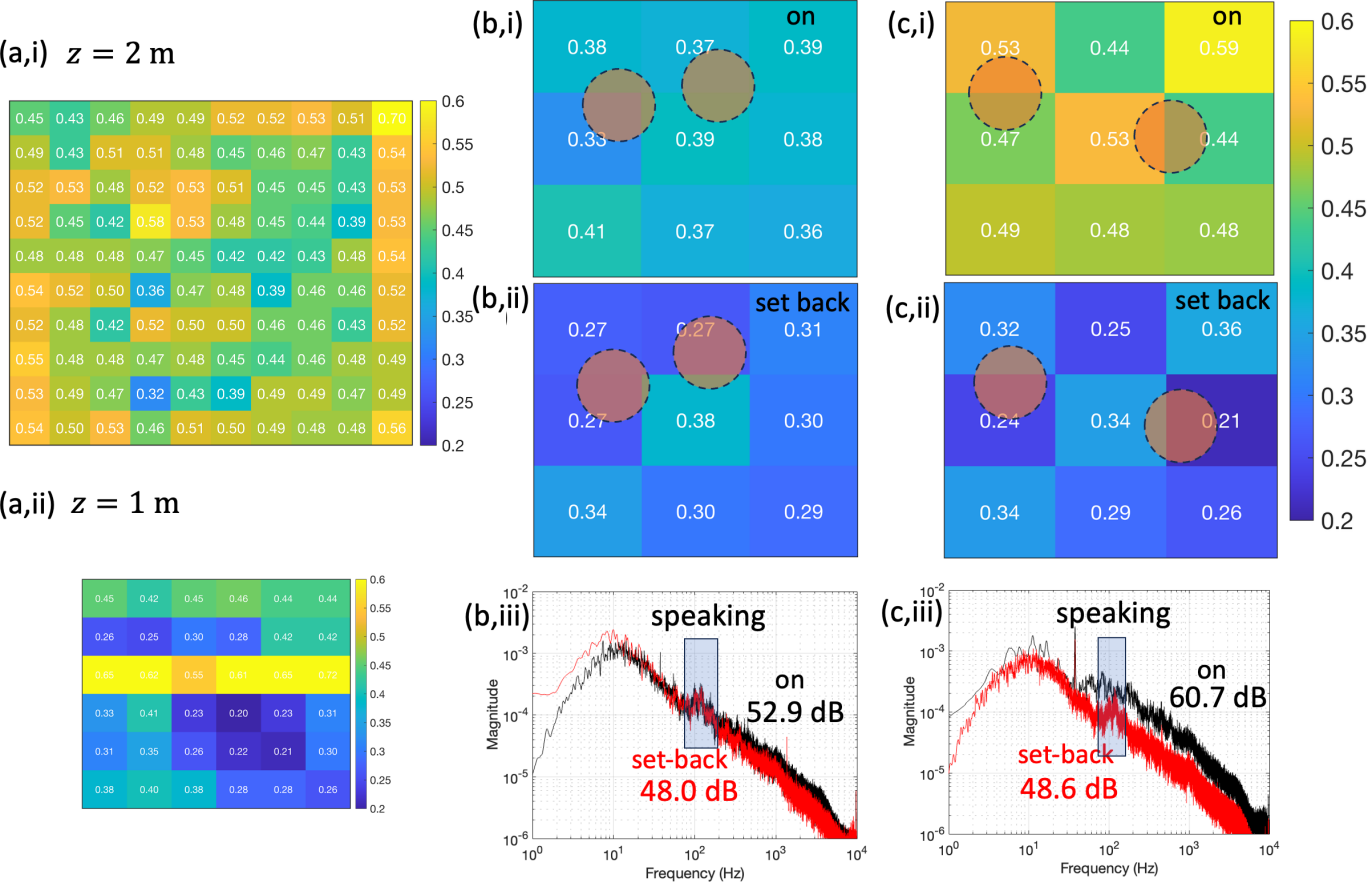
Experimental measurements of the vertical (downwards) air speed, $-u_{z}$ (in $\text{m}\;{\text{s}}^{-1}$) for LFV under different conditions. The velocity scale is the same in each figure. In (a), a typical validation report is shown for the z=2 m and z=1 m (in the inner region). In (b,c), the vertical air speed is shown at z=2 m, measured at the hood edge and centre and averaged over five measurements, with the lamp locations approximately indicated by circles. The results are shown under full mode and setback (or conventional operation) for (i) and (ii), respectively. The sound intensity and frequency distribution are shown for full (on) and setback operation.

Figure 9 shows experimental measurements of the air flow and sound signature beneath the hood of a LFV system, highlighting the challenges in air flow management. In figure 9a(i,ii), a typical validation report for the vertical velocity is shown for z=1,2 m, and highlights the strong spatial variation in $u_{z}$ and the excess ventilation delivered compared with (2.6). Figure 9b,c shows the vertical air speed in measured using Testo 405i and averaged over four measurements (separated by 5 min). The results contrast the effect of full operation and setback operation of two LFV systems, with the air-handling unit delivering in excess of 65 ACH in setback operation. Lighting has a strong influence on the air flow within the canopy and this is evident in figure 9b,c (and figure 8b) where the position of the lights are approximately indicated. (The influence of lights is minimized during validation by pushing the lights to one side). Figure 9c shows a LFV system that is over-ventilated (with the average vertical velocity $0.49\;\text{m}\;{\text{s}}^{-1}$) by the fans in the hood. During setback, the sound intensity and spectral distribution of sound intensity are approximately the same in both theatres. During full operation, the sound intensity in the over-ventilated theatre (figure 9c,iii) is 60.7 dB due to excessive load on the overhead fans. The sound intensity in figure 9b,iii is within the acceptable range (table 2).

## 3. Theatre suite energy consumption

**Figure 10.**
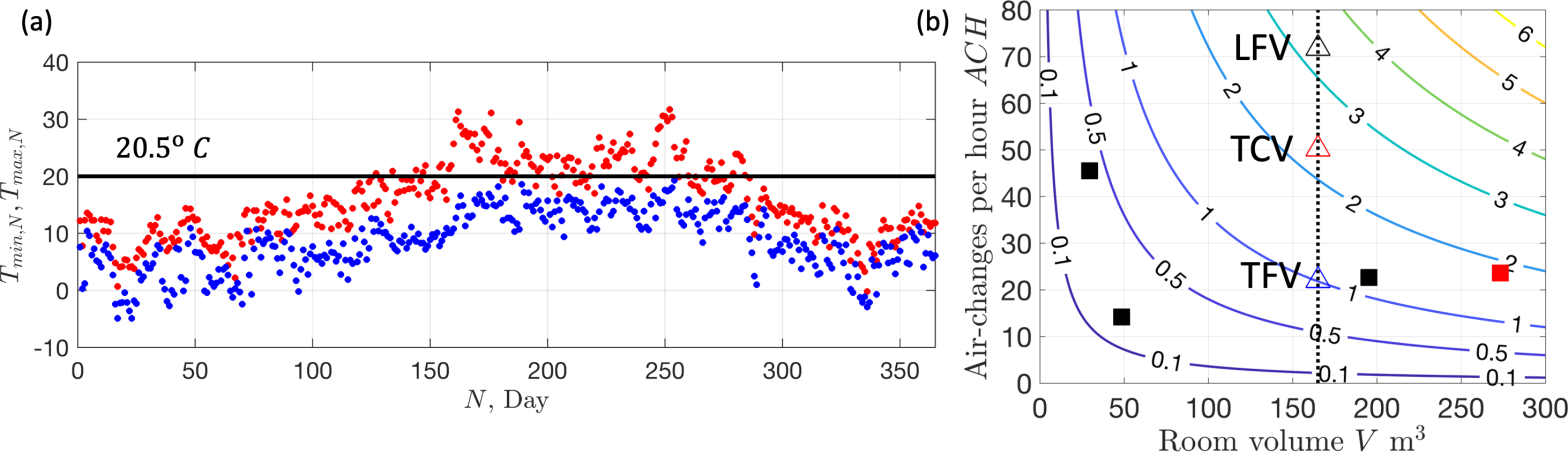
(a) The variation of the maximum and minimum temperature, on each day of the year, at Heathrow Airport in 2023. The data is taken from http://nw3weather.co.uk/. The horizontal line is a set point of $T_{sp}=20.5^{\circ }\text{C}$. (b) Scatter plot showing the prescribed ACH for different rooms with isocontours correspond to volume flux (in m^3^ s^−1^). The vertical line corresponds to a typical operating theatre ($55\;{\text{m}}^{2}$, H=3 m) with the triangles correspond to the ACH for TFV, TCV and LCV (see table 1). The black filled squares correspond to the theatre, anaesthetic room and preparation room (figure 3b) with the red square corresponding to the whole theatre suite.

The thermal challenge of delivering clean fresh air (via the ventilation system) into a theatre suite is highlighted in figure 10a, which shows the maximum and minimum daily outside air temperatures (OAT) (recorded at Heathrow Airport, typical of the southeast of the UK) along with a target temperature of $20.5^{\circ }\text{C}$, which is typically set as the supply theatre room temperature. In the UK, most of the time fresh air is heated, with cooling typically performed in summer, to bring the humidity below 70% or during periods of high thermal loads. The air is delivered at a volumetric flow rate that depends on room ACH requirements and size (see figure 10b). Theatre suite energy consumption comes from the equipment used within the space and the energy expended in conditioning and supplying air into the suite. The total energy depends on the efficiency of each subsystem, ventilation system and mode of operation. In this section, we develop a series of mathematical models to estimate energy demand, costs and CO_2_e across different AHU and ventilation modalities.

### 3.1. Air handling unit

**Figure 11.**
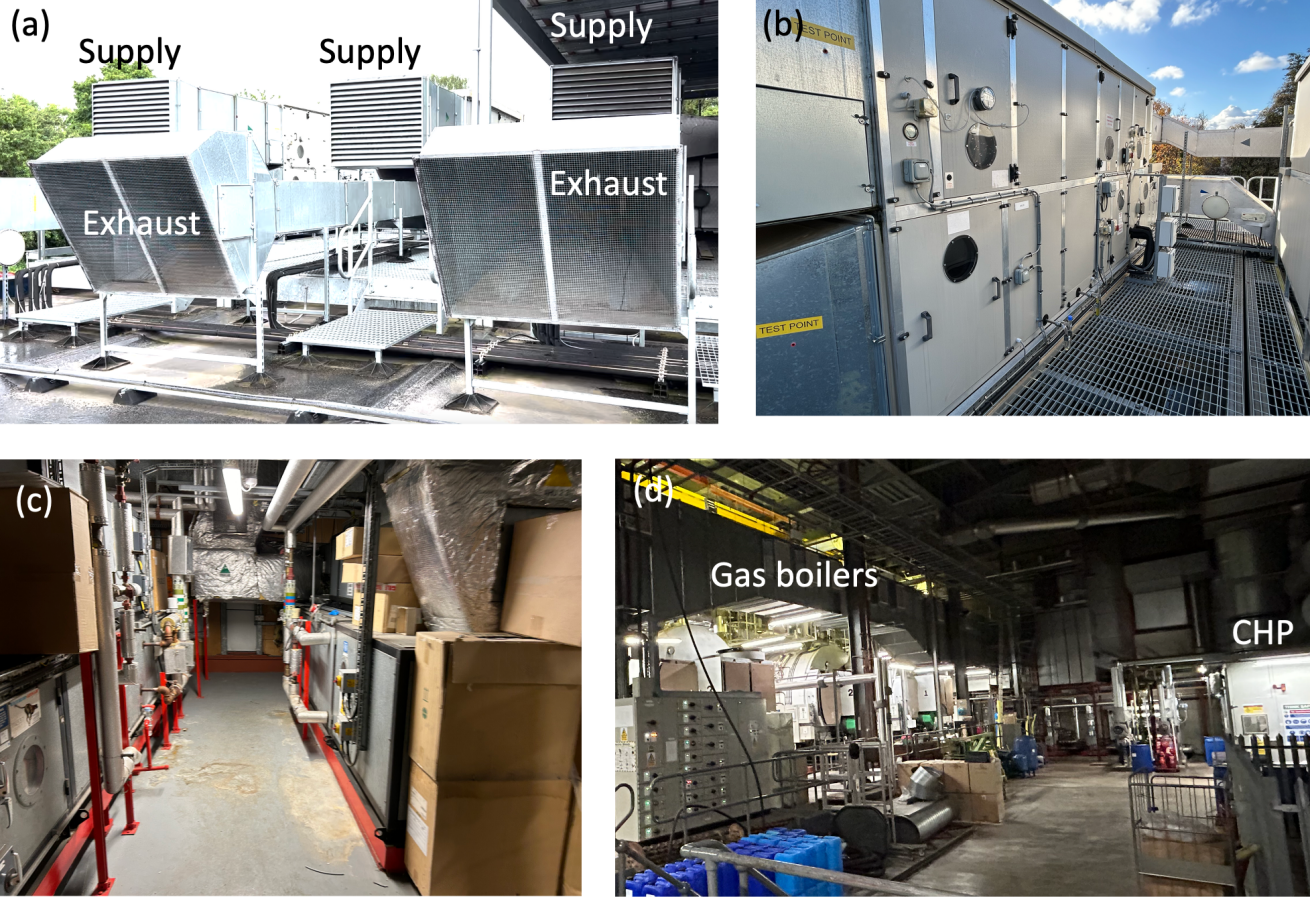
Photographs of typical air-handling units. In (a), the supply and exhaust vents of the externally mounted AHU of Theatres 11 and 12 (Whipps Cross) and in (b), the inspection hatches, drain ports and manual/electronic manometers placed across filters can be seen. The systems (a,b) are wholly electric with a stacked arrangement. A typical steam-heated AHU system is shown with the chiller and steam pipes for the battery heater are visible in (c). The traditional gas-powered boilers and a combined heat and power (CHP) system are shown in (d) (Whipps Cross Energy Centre).

AHUs supply clean air to the operating theatre suite at a specified temperature and volumetric flow rate. It comprises mechanical and electrical components that are monitored and controlled by a building management system (BMS), which combines logic programming and system sensors for precise regulation. AHUs can be installed on the rooftops (figure 11a,b), while in newer, multi-story hospitals, they are often placed on a single floor, with exhaust and supply ducts connected to the building’s exterior. To facilitate maintenance, AHUs are designed with multiple inspection ports, lighting and mechanical pressure drop meters that monitor filter conditions. Double-stacked AHU configurations (figure 11b), with the supply and exhaust section in parallel, are easier to integrate with a heat recovery system; when this is not achievable, heat recovery is achieved with a runaround coil. Generally, sensible heat recovery is used in the UK, reducing the chance of mixing the exhaust and supply air.

**Figure 12.**
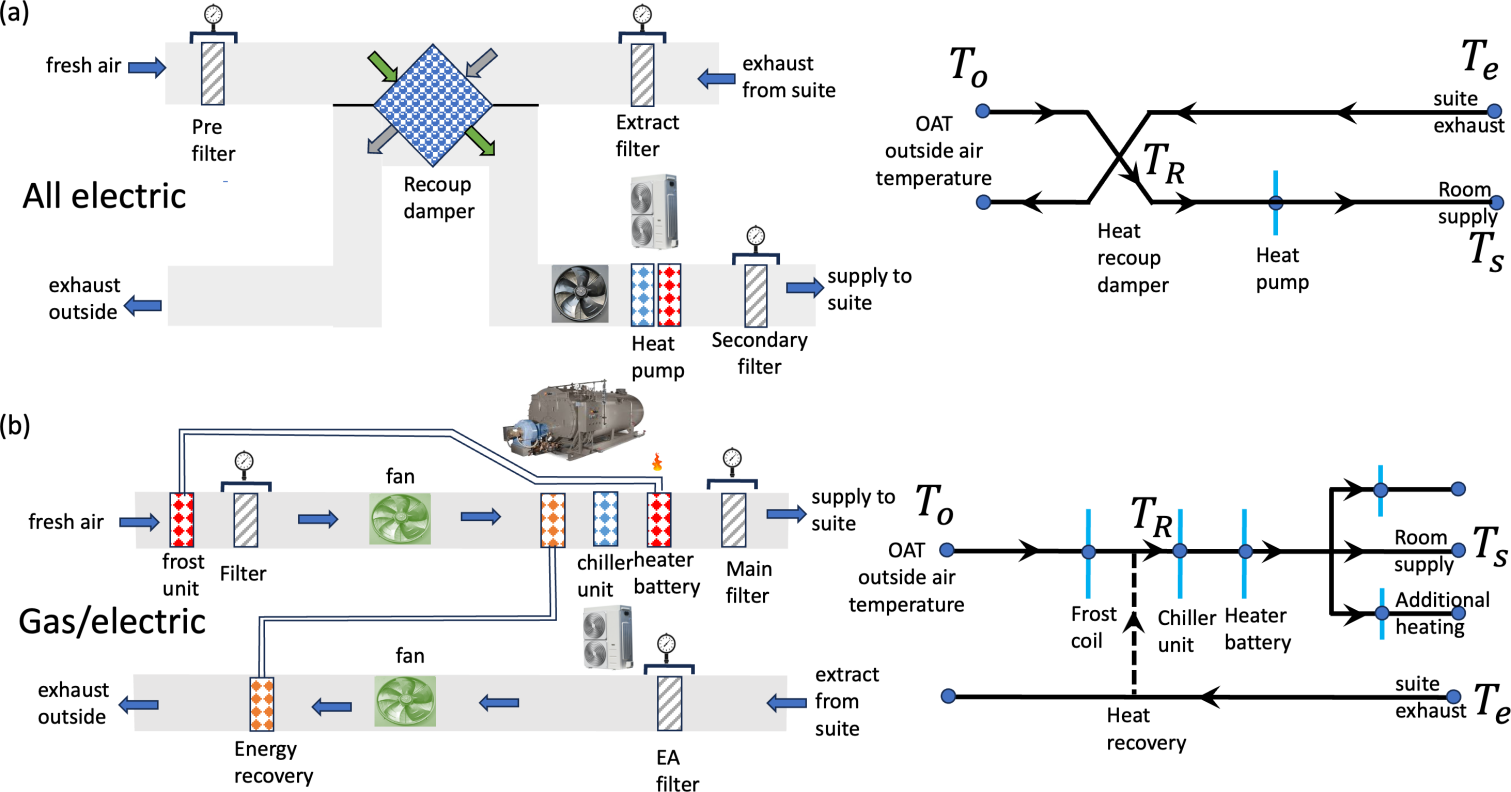
A schematic representation of two contrasting air handling units (AHUs) is shown along with a nodal representation of the air pathway. In (a), a typical all-electric system (e.g. Theatre 12, Whipps Cross Hospital) with heat recovery via a recoup damper is shown. In (b), a typical mixed gas/electric AHU system is shown, with heating supplied from a gas boiler and cooling from a chiller unit. The heat recovery comes from a recirculating loop and coils. In these examples, the supply and exhaust air during heat recovery remain separate.


The topology of the air route through the handling unit falls into two categories. When heating is provided by heat pumps (figure 12a), the entire heating-cooling systems are integrated together. When heating is provided by a hot water loop from a boiler or combined heat and power (combined heat and power CHP) (figure 12b), the air sequentially passes through frost protection, heat recovery, cooling coils and a heating battery. Both systems incorporate heat recovery, which can take one of two forms: sensible heat recovery—involving a heat exchanger that transfers heat without mass exchange, typically via a circulating water loop or a recoup damper—or latent heat recovery, where both heat and moisture are exchanged between the exhaust air and fresh supply air, commonly using a thermal wheel or a damper with a grille. The latter is characteristic of a TCV system. The enthalpy of the air, h, depends on both temperature and humidity with

(3.1) $$\begin{array}{lcr} & h=c_{p}T+\phi (L+c_{pw}T), & \end{array}$$

where $c_{p}=1.005\;\text{k}\text{J}\;\text{k}{\text{g}}^{-1}{\text{K}}^{-1}$ is the specific heat capacity of dry air, ϕ is the humidity ratio (kg of water vapour per kg of dry air), $L=2501\;\text{k}\text{J}\;\text{k}{\text{g}}^{-1}$ is the latent heat of vaporization of water, $c_{pw}=1.86\;\text{k}\text{J}\;\text{k}{\text{g}}^{-1}\;{\text{K}}^{-1}$ is the specific heat capacity of water vapour and air density is $\rho =1.15\;\text{k}\text{g}\;{\text{m}}^{-3}$. The mass loading ϕ varies from 0.00302 to 0.00721 for 40% RH and temperature varying from 10 to $20^{\circ }\text{C}$. The change in heat flux through the theatre suite is related to the heating (from people and equipment), P(t,N), which depends on time and day, in the space through


(3.2) $$\dot{m}h_{r}=\dot{m}h_{s}+P(t,N),$$

where $\dot{m}$ is the mass flux through the AHU,


(3.3) $$\begin{array}{lcr} & \dot{m}=\rho Q_{Ts}, & \end{array}$$

(see the caption in table 2 for typical values). Morgenstern *et al*. [13] measured theatre electrical consumption to be 10 $\text{W}\;{\text{m}}^{-2}$ rising to 15 $\text{W}\;{\text{m}}^{-2}$ during daytime period, corresponding to approximately 550 to 825 W (for a theatre room of 55 ${\text{m}}^{2}$). Adults generate approximately 100 W of thermal energy [27] and it is typical for the space to consist of 4 to 15 staff (for the most complex cases); anaesthetic equipment consumes approximately 100 W. The value of P can vary from 550 to 2425 W during periods of operation (with an adult patient). The heat flux required to condition the air supplied to the suite to a temperature $T_{s}$ (from (3.1)) is


(3.4) $$\begin{array}{lcr} & e_{h}=\dot{m}(h_{s}-h_{R}). & \end{array}$$

When sensible heat is recovered and the humidity ratio is unchanged by the AHU, the air temperature post-recovery, $T_{R}$, is raised by the extract air (temperature $T_{e}$) from the outside air temperature, $T_{o}$, to


(3.5) $$\begin{array}{lcr} & T_{R}=T_{o}+\alpha (T_{e}-T_{o}), & \end{array}$$

where the recovery efficiency is α (typically α∼ 0.5–0.7). When heat recovery is achieved by mixing exhaust air with a fraction r of fresh air and the humidity ratio differences are small, the temperature changes are described by (3.5) with r=1−α (r≥0.2 [26]). When the change in ϕ is small, the total heat flux required to condition the air can be estimated by combining (3.1–3.5) to give


(3.6) $$\begin{array}{lcr} & e_{h}(t,N)=\rho c_{p}Q_{Ts}\left(1+\phi \frac{c_{pw}}{c_{p}}\right)\left(T_{r}-(1-\alpha )T_{o}-\alpha T_{e}\right)-P(t,N). & \end{array}$$

**Figure 13.**
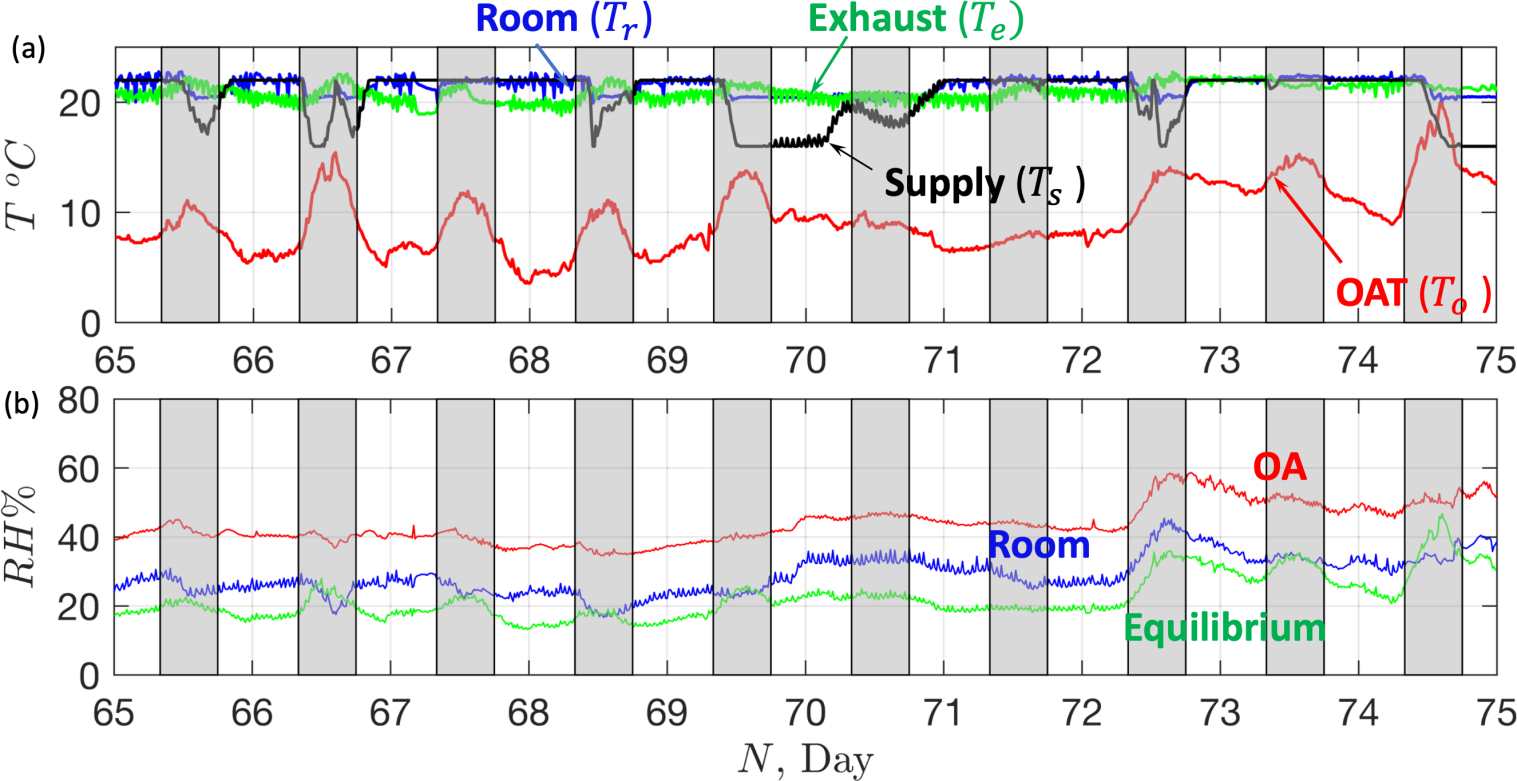
Variation of the (a) temperature and (b) humidity in a fully electric AHU over a 10-day period starting on 5 March 2024 (N=65). In (a), the OAT ($T_{o}$) is contrasted with the room ($T_{r}$), extract ($T_{e}$) and supply air temperature ($T_{s}$). In (b), the OA humidity is contrasted with the room humidity. The green curve corresponds to the prediction of room humidity based on raising the external air temperature to the room temperature. The grey rectangles correspond to the period of time extending from 7.00 to 19.00.

When $e_{h}>0$, heat is being added to the air while for $e_{h}<0$, cooling is required. Figure 13a shows a time series of temperature for a fully electric AHU over a period of 10 days (starting 5 March 2024, N=65) for an AHU servicing Whipps Cross Hospital (Theatre 12, see figures 2, 3a, 10a and 11a). The grey boxes correspond to the period 7.00 to 19.00. During the working day, the BMS is searching to maintain the room temperature at $T_{r}=20.5^{\circ }\text{C}$ during which exhaust temperature tends to be higher (at $22^{\circ }\text{C}$) and the supply temperature is lower. The temperature difference between the OAT and room temperature varies between $5^{\circ }\text{C}$ and $15^{\circ }\text{C}$. At night, when the room’s thermal load is low, the difference between the room and supply air temperature is small. During the day, cooler air is supplied to overcome the thermal load in the suite. The extract air temperature is lower, in this case, because it draws cooler air from the corridor. During periods of high activity and increased thermal loads, the supply temperature decreases to $16^{\circ }\text{C}$, which is the minimum temperature set by the BMS.

### 3.2. Air handling unit energy demand

The AHU energy demand comes from the thermal energy required to condition the air to the desired room temperature and the electrical fans to drive the air flow. The contribution to the thermal energy comes from three different cycles—(i) cooling which can come from a heat pump integrated into the AHU (figure 11a) or serviced by a large chiller unit (again, with a set of heat pumps; figure 11b). (ii) heating from a heat pump or delivered via a heat exchanger with a hot water supply heated in a boiler, and (iii) reduction of humidity in which air is chilled below the dew point and then reheated. Fans tend to run with a constant load which is far less variable than the thermal load.

In the analysis, the mass fraction of water vapour is unchanged, the total thermal energy difference between the ambient temperature and the set point (3.6) is determined by integrating the thermal power (3.6) over the period of the day during which the AHU is in operation,


(3.7) $$\begin{array}{lcr} & E_{h}(N)=\int_{t_{on}}\left|e_{h}(t,N)\right|dt. & \end{array}$$

The model is driven by the OAT, $T_{o}$, which changes over time and depends on the geographical area. The OAT varies between a maximum $T_{\text{max},N}$ and $T_{\text{min},N}$*,* for each day N of the year (N=1,...,365) which can be expressed in terms of time t(s) from the start of the day


(3.8) $$T_{o}(t,N)=T_{\text{min},N}+(T_{\text{max},N}-T_{\text{min},N})f\left(t,N\right).$$

The OAT is strongly influenced by solar gain and tends to a minimum during the night and a maximum during the day. A number of empirical fits could be applied; here the closure chosen is $f\left(t\right)=\frac{1-\sin\left(\frac{\pi (t-12)}{24}\right)}{2}$, which satisfies f(0)=0 and f(t=12 h)=1.

The daily fan energy consumption on day N of the year is the product of the period of operation with the fan power for the supply ($W_{s}$), extract ($W_{e}$) and additional fans that are required for LFV ($W_{\text{LFV}}$),


(3.9) $$E_{f}(N)=(t_{\text{on}}(N))(W_{s}+W_{e}+W_{\text{LFV}}).$$

The electrical fan power W is proportional to the mechanical power required to drive a volumetric flow Q and pressure differential $\Delta p_{f}$, $W=Q\Delta p_{f}/\eta$, where η is the efficiency. In general, W=Q/SFP where the specific fan power (*SFP*) depends on the presence of HEPA filters and resistance in the air flow, and a value of 2000 $\text{W}\;{\text{m}}^{-3}\;\text{s}$ is typical of AHU for existing builds of non-domestic ventilation systems with heating, cooling and heat recovery [28, table 23].

The total energy demand differs from thermal energy costs due to variations in system efficiency. Boilers typically have a coefficient of performance (COP) ranging from 0.7 to 0.83 [29], reflecting the conversion of thermal energy from combustion into usable heat. However, thermal losses occur in piping, primarily due to radiant heat and conductive losses, especially in large hospital sites and centralized energy centres. Pump energy costs are influenced by pipe length, biofouling and even when the hot water supply is diverted through a valve—such as when the AHU is not drawing heat—the pump continues to operate. Chiller units, which often service multiple systems, typically have a COP between 2.5 and 4.5, while the COP of an air-source heat pump is approximately 2.5 [29], though integration within systems brings this down to about 2.0. The total energy demand is now estimated—the calculations provide a lower bound to the approximate cost and serve to demonstrate the consequence of a change in use.

The yearly energy consumption is evaluated by summing the daily consumption,


(3.10) $$\begin{array}{lcr} & E_{T,h}=\sum_{N=1}^{365}E_{h}(N),\;E_{T,f}=\sum_{N=1}^{365}E_{f}(N),\;E_{T}=E_{T,h}+E_{T,f}. & \end{array}$$

The units of $E_{T,h}$, $E_{T,f}$ and $E_{T}$ are J but the results are converted to MW h by division with $3.6\times 10^{9}$.

### 3.3. Energy estimates for three theatres

The annual energy consumption of the three theatre types (figure 2) is assessed under identical thermal load, suite layout and room temperature, running in continuous operation, by evaluating (3.10) using (3.7) and (3.9). The model requires setting $T_{e}$, $T_{r}$ and P. Here, the theatre temperature is set as $T_{r}=20.5^{o}\text{C}$ during the period 8.00 to 18.00, and $T_{r}=22^{\circ }\text{C}$ outside this period. Here, the temperature of the extract air, drawn mainly from the corridor outside the theatre (see figure 13) is set at $T_{e}=21^{\circ }\text{C}$. The thermal power, P, is set to be 1625 W (corresponding to seven staff and an adult patient) during the working day (7.00 to 19.00) and 550 W outside this period.

**Figure 14.**
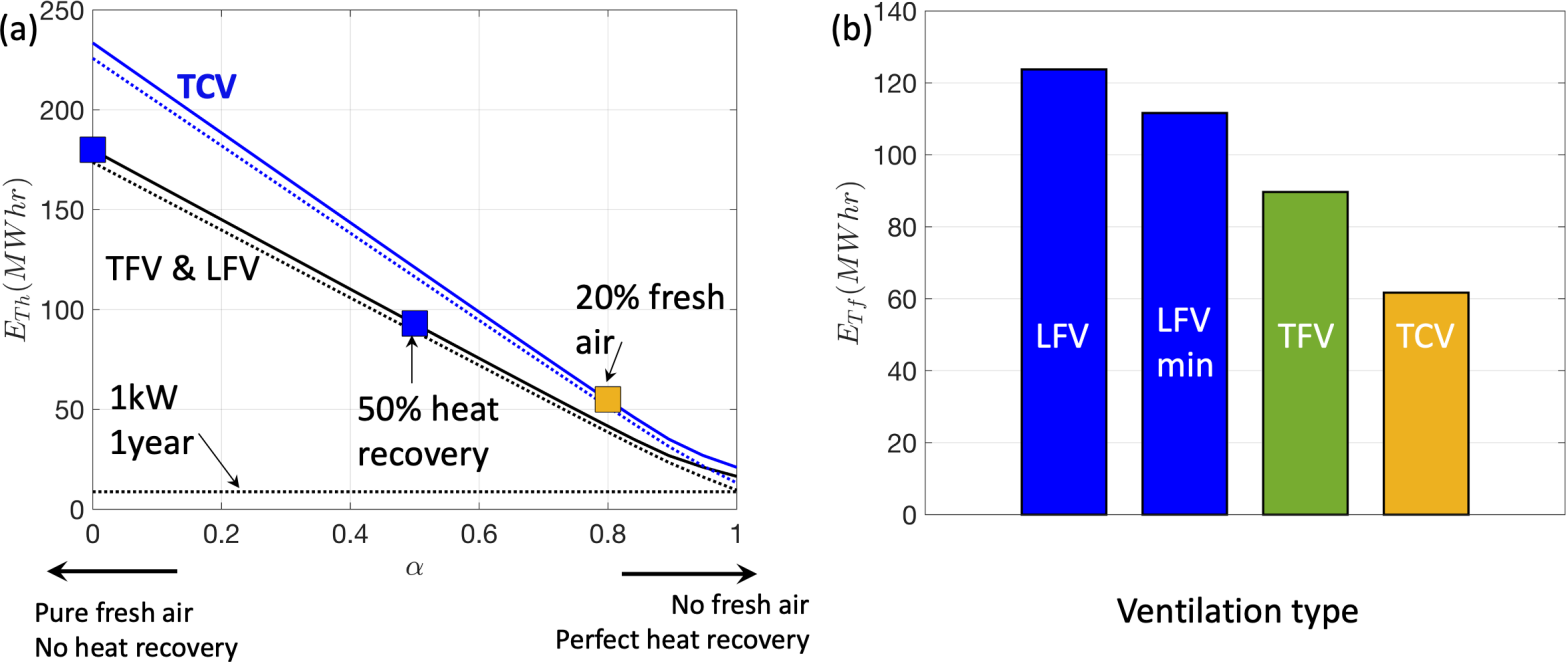
(a) The thermal heat energy demand, $E_{Th}$, for one year of continuous load is plotted as a function of air freshness (1−α) for different ventilation systems that operate in full ventilation mode continuously. The full curve corresponds to $E_{Th}$ with the dashed curve corresponds to the heating contribution. The horizontal line shows the energy corresponding to 1 kW in continuous mode of operation. (b) The fan energy demand, $E_{Tf}$, is shown for the different ventilation systems (see table 1). The energy consumption for LFV is estimated using ${\bar{u}}_{z}=-0.45\;\text{m}\;{\text{s}}^{-1}$ and ${\bar{u}}_{z}=-0.38\;\text{m}\;{\text{s}}^{-1}$ (which is labelled as LFV (min)). The estimates are based on an extract temperature $T_{e}=20.5^{\circ }\text{C}$ during the working period and $T_{r}=22^{\circ }\text{C}$ during setback and at night. The thermal power P is set at 550 W outside the working period and 1625 W (corresponding to seven adults in theatre) during the working day.

The annual thermal energy evaluated using (3.7–3.10) is shown in figure 14a as a function of 1−α. The horizontal line in figure 14a indicates the energy consumed by a 1 kW device running continuously for one year. The thermal energy curves for LFV and TFV are identical because the ACH passing through the AHU is the same in both cases, but the net volume flux is higher for TCV. The symbols correspond to typical flow exchange that corresponds to TFV or LFV (without any heat recovery), LFV with heat recovery (α=0.5) and TCV (with 20% fresh air, r=0.8). Figure 14b shows the fan energy demand for continuous operation for the different ventilation types. A comparison is made between the fan energy costs of LFV running at the 0.45 $\text{m}\;{\text{s}}^{-1}$ and one running at 0.38 $\text{m}\;{\text{s}}^{-1}$ (labelled as LFV min). As expected, a higher volume flow rate, due to over-ventilation, leads to greater energy costs.

## 4. Improving energy efficiency of theatre suites

The three key themes—suite architecture (§2.1), ventilation strategy (§§2.2 and 2.3) and AHU systems (§3.1)—have been reviewed and evaluated. By integrating the analyses from §2,3, the potential for reducing both theatre suite energy consumption and CO_2_e emissions is systematically assessed.

### 4.1. Theatre suite design and layout

The traditional UK theatre suite includes both an anaesthetic room and a scrub room. The primary rationale for an anaesthetic room is that it serves as a staging area for patient preparation, facilitating a faster turnaround between surgeries. However, this set-up incurs additional costs due to the duplication of anaesthetic equipment, drugs and services, as illustrated in the photograph of a typical anaesthetic room (figure 3b). In contrast, most European hospitals do not have dedicated anaesthetic rooms, instead preparing patients directly in the operating theatre. The ventilation burden from the anaesthetic room accounts for approximately 0.2 ${\text{m}}^{3}\;{\text{s}}^{-1}$ (figure 3b), representing around 10% of the total energy costs, as estimated from (3.7).

There is significant variation in theatre-scrub room configurations (see figure 1), with connections to the operating theatre typically consisting of a corridor, annex or a corridor with a connecting door. A net outward air flow through the scrub room into the corridor promotes a pressure differential, directing air movement through the pressure stabilizers. However, the effectiveness of this air flow in reducing droplet spread (figure 3a), given the proximity of the scrub area to the theatre space, remains unclear. The inclusion of a connecting door provides an additional barrier against the dispersion of contaminants into the theatre, particularly in sliding door arrangements.

In Europe, heightened concerns regarding waterborne pathogens have led to the relocation of scrub areas from the theatre to the clean corridor, as seen in hospitals such as Sint Maartenskliniek. The scrub area is generally not included in the theatre’s ventilated volume, and since the ventilation strategy follows a through-flow approach, its impact on overall ventilation demand is expected to remain unchanged.

### 4.2. Over-ventilation

Annual ventilation validation ensures that all ventilation in hospital operating rooms complies with national guidelines. For LFV, with a hood and within a confined space, the flow unsteadiness and mixing reduce the average velocity at the edge of the inner region (constraint (2.6b)). To satisfy this minimum vertical air speed, the air flow rate is typically increased, as illustrated in figure 9a,iii. Confinement effects reduce the decay in (2.1) but this leads to a significant increase in flow unsteadiness due to the shear layers interacting with the walls and the exhaust (see figure 6b,i). The over-ventilation increases the total energy costs, as shown in figure 14b, which contrasts the energy consumption of LFV running under a typical over-ventilation state with the LFV running with the minimum volume flow rate. In addition, over-ventilation can also increase the theatre noise (see figure 9b,ciii).

### 4.3. Ventilation system

LFV originally emerged from consideration of the orthopedic surgery, which has the potential to generate aerosols due to cutting, drilling and dispersing material via fast-moving air patterns throughout a space. The original intention of the laminar flow ventilation was to pass ultra-clean air over the patient. The ventilation validation of LFV is devised to show the coherence of the shear layer and the limited entrainment in the absence of the surgical table, patient and surgical staff. Both LFV and TCV create a vertical flow in an inner region characterized by a small relative RMS velocity. The flow in the inner region is disrupted by the presence of the surgical table, which creates a local straining flow and the presence of staff and theatre lights which create recirculating regions. Any material that becomes dispersed into the air is potentially swept back towards the wound and this effect becomes much stronger as the vertical speed increases, leading to a greater potential for surgical site infections (SSI) for LFV compared with TCV. In contrast, the TFV focuses on removing and diluting airborne contamination, which occurs through a horizontal flow that sweeps across the surgical site. The intrinsic unsteadiness in turbulent flow ventilation serves to enhance the mixing near the surgical site.

Bischoff *et al*. [30] reported a meta-analysis of the link between the occurrence of SSI, the type of surgery (orthopedic, abdominal and vascular) and ventilation type (TFV and LFV). The analysis showed no benefit for laminar air flow compared with conventional turbulent ventilation of the operating room in reducing the risk of SSIs in total hip and knee arthroplasties, abdominal surgery, and questioned the need to install LFV in theatres, a point supported by [31]. The disruption of the ultra-clean space by lights, people and equipment may provide an explanation for the similar outcomes for SSIs for LFV and TFV. TCV has an advantage because the air is partially buoyancy-driven and less susceptible to disruption by the presence of a person or light.

From an energy perspective, LFV typically uses 20% more fan energy than TCV, simply owing to having a higher volumetric flow rate. A typical TCV configuration has 20% fresh air recycling, which has a greater efficiency than a typical LFV with a heat recovery system in the UK. In addition, TCV is also quieter than LFV because the air is driven wholly by the AHU and not by fans in the hood. Figure 14a underlines the importance of energy recovery systems to reduce the energy burden—with the three boxes denoting hospitals with LFV and no energy recovery, a heat recovery system and a mixer delivering 20% clear air. The reduction in costs is approximately in proportion to the efficiency of heat recovey or fresh-air dilution fraction.

### 4.4. Setback and switch-off

Theatre ventilation systems are expected to utilize the minimum energy. This means that switching the AHU system off, when not required, is the most energy-efficient policy ([16], §6.1), with putting the system in setback, the next best option. Setback requires running the AHU fan at a reduced speed of 40% of the maximum fan speed (to prevent flow stall past the fan blades); for LFV, the hood fans are turned off during setback. The operation of setback of the theatre is usually timetabled by the BMS system, but it is common for this state to be permanently switched to full operation when passive infra-red (PIR) sensor’s are triggered (for instance, when the suite is being cleaned). The state of the ventilation system is visually indicated on the surgeons panel, but there is often confusion among staff about whether the system is in setback and concern about when it is in this state. Driven by the NetZero target, there is a strong incentive to switch off theatres that deal with scheduled operations and when not in use [32].

**Figure 15.**
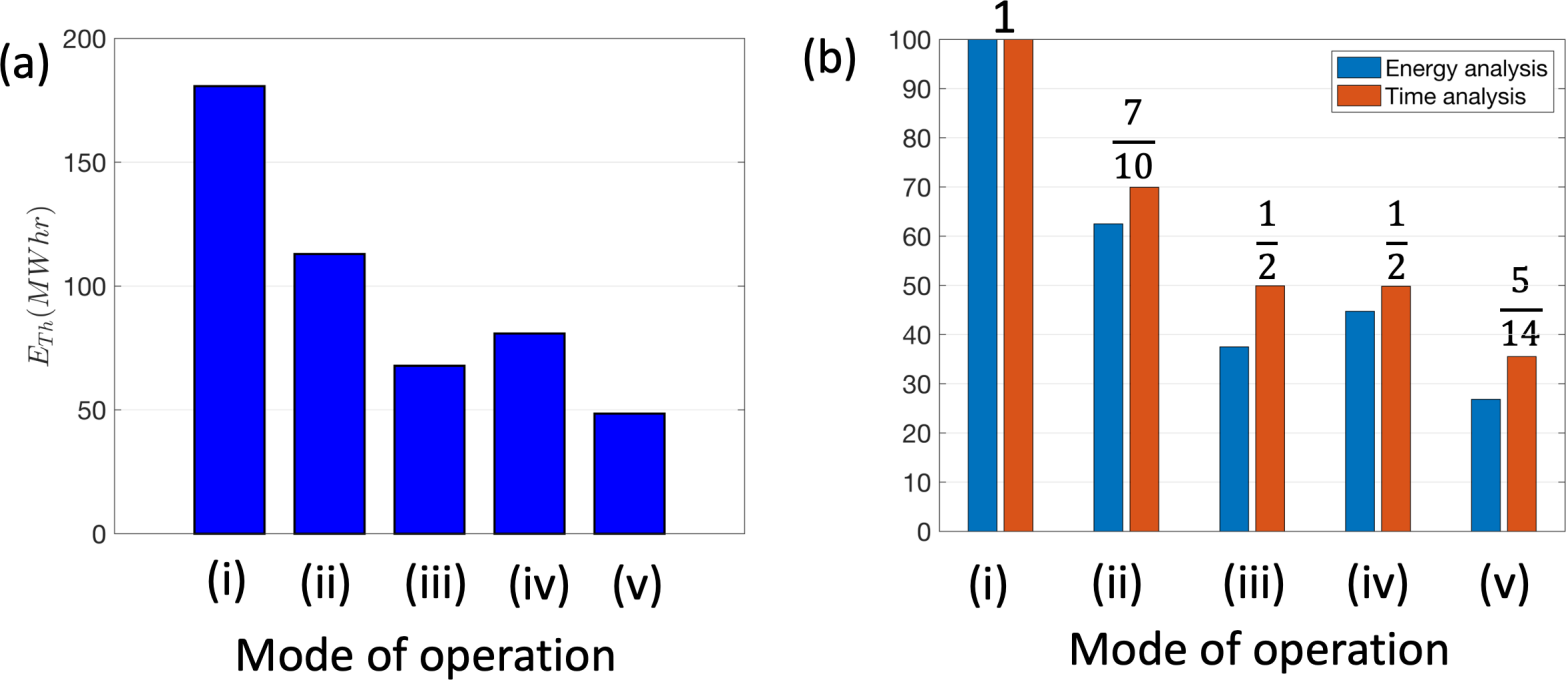
(a) The influence of the mode of operation on the thermal energy consumption. This case corresponds to a LFV with air supplied from an AHU that is heated by a gas-boiler. The working day is defined to extend from 7.00 to 19.00, during which the operating theatre ventilation is set to full operation. The modes of operation are (i) continuous, (ii) set back outside working day, (iii) off outside working day, (iv) set back outside the working day and w/e and (v) off outside working day and weekend. In (b), ratio between the thermal energy consumption compared with the benchmark case of full operation of a gas-boiler heated AHU is shown. A comparison is given between a fractional time estimate and that based on integrating consumption over time.

A comparative analysis of the thermal energy demand for a TFV (with $Q_{Ts}=1.86\;{\text{m}}^{3}{\text{s}}^{-1}$) is made in figure 15a for different modes of operation corresponding to continuous operation, setback operation outside the working day, switch-off outside the working day, set back during the evening and weekend, and switch-off outside the working day and at the weekend. The working day was defined here, purely as illustration, as 7.00 to 19.00, corresponding to $t_{on}=12\;\text{h}$. The thermal heat energy is evaluated by integrating (3.7) over the period during which the AHU is on and weighting the air flow with a factor of 0.4 during setback periods. In figure 15b, a comparison is made between the integral analysis and one based on fractional reduction in the use of the AHU (in time and load). The approximate model provides a leading order estimate of the reduction in consumption, but tends to underestimate the thermal energy analysis because the energy consumption is higher during the evening when the OAT is lower. A summary of the total energy costs, for the different modes of operation, are listed in table 3. The analysis highlights the significant reductions in thermal energy consumption that can be achieved when the AHU is switched off during periods of theatre non-use.

**Table 3.** Table showing a comparative (minimum) annual energy, cost and CO_2_e production, associated with the ventilation of an operating theatres suite. The heat recovery estimate is based on sensible heat. The three ventilation systems are considered (TFV, LFV, TCV) along with different heating modalities.

Ventilation type	electric/gas	heat recovery	$E_{T}$	cost ( Co )	CO_2_e
		α	( MW h )	( £000 )	( $\text{tonnes}\;{\text{yr}}^{-1}$ )
**TFV—full power**	**mixed**	**0**	**357**	**38**	**67**
TFV—setback evenings	mixed	0	225	25	42
TFV—off evenings	mixed	0	145	16	27
TFV—setback evenings and w/e	mixed	0	104	12	19
TFV—full power	mixed	0.5	212	27	40
TFV— off evenings and w/e	mixed	0.5	62	9	12
**TFV— full power**	**electric**	**0.5**	**108**	**29**	**21**
TFV—setback evenings	electric	0.5	71	19	14
TFV—off evenings and w/e	electric	0.5	34	9	7
**LFV—full power**	**mixed**	**0**	**408**	**51**	**76**
LFV—setback evenings	mixed	0	261	34	49
LFV—off evenings and w/e	mixed	0	122	17	23
LFV—off evenings	mixed	0.5	80	14	15
**LFV—full power**	**electric**	**0.5**	**158**	**43**	**31**
LFV—off evenings and w/e	electric	0.5	52	14	10
**TCV—full power**	**electric**	**0.8**	**122**	**33**	**24**
TCV—setback evenings	electric	0.8	81	22	16
TCV—off evenings and w/e	electric	0.8	40	11	8

There are two concerns about switching off the AHU which are related to the potential for contamination and its link to patient safety. Dettenkofer [33] examined the CFU load and airborne particle counts during off-duty periods and observed that the particle count and microbial contamination diminished to acceptable levels shortly after the system is restarted. The second concern is that switching off the AHU may lead to the pressure differentials between adjacent rooms, changing sign and should be assessed. Pressure differentials in complex spaces are greatly affected by the topology of the network connections between those spaces and the effects can be non-local. The outbreak of SARS in Amoy Gardens during 2003 in Hong Kong ([34,35]) highlights this effect with a non-local pathway of infection caused by flow induced by cross-winds and between living spaces caused by pressure differentials.

**Figure 16.**
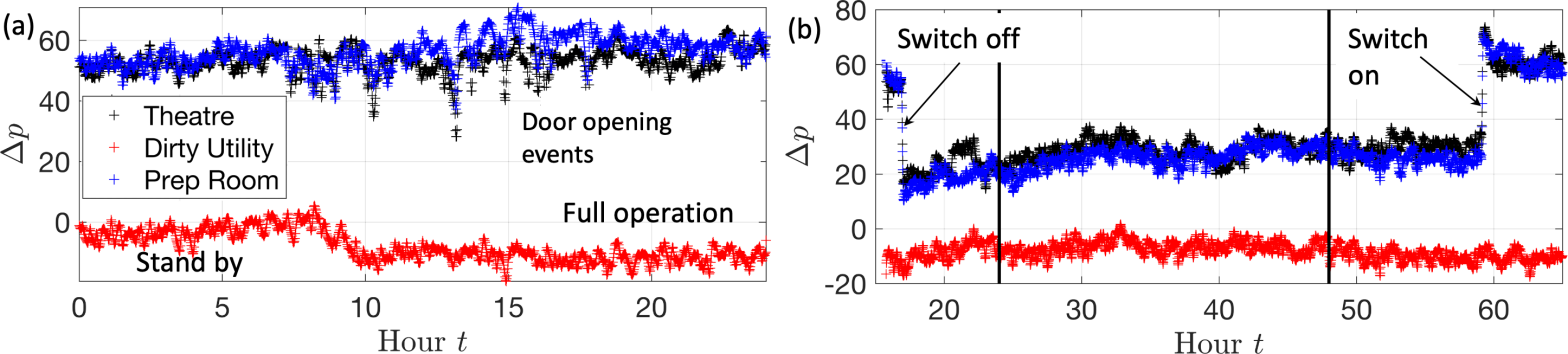
The pressure differentials between the rooms and the hospital bay (taken as a proxy for corridor pressure) is shown as a function of time for Theatre 12 at Whipps Cross Hospital; the legend identifies the pressure differential in the theatre, dirty utility and preparation room. In (a), the pressure differential on 18 March 2024, shows a normal process of operation with t=0 corresponding to midnight. In (b), the variation of the pressure differentials with time are shown for the period during which the AHU is switched off and then on.

The consequence of switching off an AHU on the pressure differential between rooms was analysed experimentally by setting up wireless absolute pressure sensors at Whipps Cross Hospital in the Theatre 12 suite; the data from the monitors were smoothed using a median filter and then logged on a University College London server. Figure 2a shows the position of the sensors (denoted by the letter S) within the theatre, dirty utility and the preparation room. The pressure sensor (24 bit, MicroPressure MPR Series Honeywell) was located in a corridor (off plan) to act as a reference measurement. Figure 16a shows the pressure differential between the theatre, dirty room and preparation room during normal operation. The pressure in the dirty utility is less than the corridor and drops by −10 Pa during normal operation. The pressure in the preparation room is approximately 10 Pa above the theatre, with the theatre approximately 50 Pa above the corridor. When the AHU is switched off, the sign of the pressure differentials are unchanged, but the magnitude is reduced. In this configuration, the corridor extract from Theatre 11 appears to drive a weak flow through the theatre space in Theatre 12, and this is enhanced by flow separation due to wind past the roof-mounted exhaust vent (figure 11a) that draws air through the theatre. When the AHU is switched off, the pressure differential will be set by the flow through the gaps around the door, rather than the pressure stabilizer. Overall, these insights underscore the importance of carefully managing air flow pathways in hospital ventilation strategies, particularly when implementing energy-saving measures like AHU switch-off.

The financial cost (Co) for theatre suite ventilation can be estimated from the energy demand $E_{T}$ (noting the neglect of thermal losses, air scrubbers and pumps) by separating into the total electrical $E_{e}$ and gas $E_{g}$ components, with the yearly financial cost $Co=c_{e}E_{e}+c_{g}E_{g}$, where the electricity and gas unit costs (May 2024) are $c_{e}=27\;p\;\text{k}\text{W}\;{\text{h}}^{-1}$ and $c_{g}=7\;p\;\text{k}\text{W}\;{\text{h}}^{-1}$ respectively. For a fully electric system, $E_{g}=0$ and $E_{e}=E_{Tf}+E_{Tt}/COP$, where the COP is about 2 for an electric system. For a mixed gas-electric system with heating provided by a boiler, $E_{e}=E_{Tf}$ and $E_{g}=E_{Tt}/COP$, where the COP is about 0.6 for a gas boiler. Table 3 contrasts the costs, energy consumption, CO_2_e production for LFV, TFV and TCV, for mixed (gas and electric) or fully electric systems. The benchmark costs corresponding to continuous operation are indicated in bold. The movement towards fully electric systems, with heat recovery, represents both a significant reduction in costs and CO_2_e.

A switch-off strategy, applied during the evening and weekend, would reduce expenditure by more than 70%. A number of older LFV theatres, heated by gas boilers, still operate in a continuous mode, with no heat recovery—the application of a switch off strategy will reduce annual expenditure by *£*34k and reduce CO_2_e by 53 tonnes (table 3). The most efficient fully electric LFV theatres, employing heat recovery, can reduce expenditure by *£*29k with switch-off. The TCV represents a saving, over LFV, mainly because they employ air recycling which reduces overall costs but are advantageous because of the reduction of particle load in the outer region of the operating theatre. There are approximately 3339 operating theatres in England and the potential *£*90M savings a national role-out of switching off theatres, reported by [36], looks appropriate.

Linking back to the NetZero, the reduction in energy utilization leads to a consequential reduction in CO_2_e, particularly when matched with a switch from gas-boiler heating to a fully electric system. The total CO_2_e produced can be estimated through an established methodology by linking mass to electricity and gas consumption: $m_{CO_{2}e}=\lambda_{e}E_{e}+\lambda_{g}E_{g}$. The factor $\lambda_{e}$ depends on the proportion of electricity (in $\text{k}\text{W}\;\text{h}\;{\text{yr}}^{-1}$) that is generated from non-carbon sources, but taking into account the energy losses in the transmission network, the value for the UK [36] is $\lambda_{e}=0.19338$ [37] and $\lambda_{g}=0.185$. For LFV, the estimated CO_2_e production for a gas-boiler under continuous operation is 76 tonnes ${\text{yr}}^{-1}$. By implementing a switch-off programme and introducing heat recovery systems, this total can reduce CO_2_e production by 56 $\text{tonnes}\;{\text{yr}}^{-1}$.

## 5. Conclusions

This article explores the potential for operating theatre suites to transition towards NetZero, focusing on architectural layout, ventilation system design, operational modes and air-handling unit selection. By integrating data analytics, computational fluid dynamics (CFD) simulations and energy modelling, the study quantifies the impact of system choices on energy demand.

The reduction of energy demand can be met through a combination of strategies. Switching off the theatres when not in use reduces energy consumption by a factor greater than the fractional reduction of time in use—this will be a reduction of about 60% compared with continuous operation or 30% compared with those put in setback. Older AHUs tend to be heated by gas boilers (with low thermal efficiency) without energy recovery systems. Fully electric AHUs have energy-recovery systems reducing heating costs by approximately 50%; greater reductions are afforded by recycling air. Innovations in hospital ventilation systems have been minimal since the introduction of laminar flow ventilation. However, TCV, which relies on a temperature differential and low-level extraction, shows promise by lowering CFU measurements in external spaces and minimizing recirculating vortical regions.

Achieving NetZero requires innovation and evidence-based science. This study highlights the need for cross-disciplinary and international collaboration to harness a broad spectrum of experiences and identify evolving innovations essential for tackling these societal challenges.

## Data Availability

Numerical data can be extracted from the charts or calculated using open source codes.

## Appendix A

A computational model is applied to describe the evolution of the flow field in the model operating theatres. The standard methodology is based on a detached eddy simulation using OpenFOAM v. 2112 (www.openfoam.com). The momentum equation describing the relationship between velocity $u_{i}$ and pressure p is

(A 1)
∂(ρui)∂t+∂(ρuiuj)∂xj=−∂p∂xi+∂∂xj(μe(∂ui∂xj+∂uj∂xi))+ρfi,

where $f_{i}$ is the gravitational body force acting on the flow. Thermal effects are included in the TCV, which requires the enthalpy of the air to be followed in time.

There are a large number of turbulence closure models that are suitable for describing the closure of the turbulent stresses with coupled evolution equations. Here, the detached eddy model of Spalart-Allmaras [38] was applied, which involves a RANS-type model close to walls and reduces to an LES formulation in the flow interior. This model is based on a single equation describing the evolution of the turbulent viscosity. A Spalding wall function was applied on rigid surfaces.

The model was solved using standard modules available with OpenFOAM: the *pimpleFoam* solver was applied for the TFV and LFV, while *buoyantPimpleFoam* solver was applied for the TCV. The mesh was generated using *snappyHexMesh* with an applied boundary layer mesh, refinement in the vicinity of the exhaust, lighting or supply ventilation. The mesh varied from 2.8 to 4.8 M cells. Calculations were initially run up to 120 s before the flow statistics were tracked. The assessment of the average concentration within specific volumes was performed through diagnostic routines hard-coded into the solver.
